# Advancing the integration of spatial data to map human and natural drivers on coral reefs

**DOI:** 10.1371/journal.pone.0189792

**Published:** 2018-03-01

**Authors:** Lisa M. Wedding, Joey Lecky, Jamison M. Gove, Hilary R. Walecka, Mary K. Donovan, Gareth J. Williams, Jean-Baptiste Jouffray, Larry B. Crowder, Ashley Erickson, Kim Falinski, Alan M. Friedlander, Carrie V. Kappel, John N. Kittinger, Kaylyn McCoy, Albert Norström, Magnus Nyström, Kirsten L. L. Oleson, Kostantinos A. Stamoulis, Crow White, Kimberly A. Selkoe

**Affiliations:** 1 Center for Ocean Solutions, Stanford University, Palo Alto, California, United States of America; 2 Department of Natural Resources and Environmental Management, University of Hawai‘i at Mānoa, Honolulu, Hawai‘i, United States of America; 3 Ecosystem Sciences Division, NOAA Pacific Islands Fisheries Science Center, Honolulu, Hawai‘i, United States of America; 4 Bren School of Environmental Science and Management, University of California, Santa Barbara, Santa Barbara, California, United States of America; 5 Fisheries Ecology Research Lab, Department of Biology, University of Hawai‘i at Mānoa, Honolulu, Hawai‘i, United States of America; 6 School of Ocean Sciences, Bangor University, Anglesey, United Kingdom; 7 Stockholm Resilience Centre, Stockholm University, Stockholm, Sweden; 8 Pristine Seas, National Geographic Society, Washington, DC, United States of America; 9 National Center for Ecological Analysis and Synthesis, University of California, Santa Barbara, Santa Barbara, California, United States of America; 10 Conservation International, Center for Oceans, Honolulu, Hawai‘i, United States of America; 11 Arizona State University, Center for Biodiversity Outcomes, Julie Ann Wrigley Global Institute of Sustainability, Tempe, Arizona, United States of America; 12 Global Economic Dynamics and the Biosphere Academy Programme, Royal Swedish Academy of Sciences, Stockholm, Sweden; 13 Curtin University, Department of Environment and Agriculture, Perth, Australia; 14 Biological Sciences Department, California Polytechnic State University, San Luis Obispo, California, United States of America; 15 Hawai‘i Institute of Marine Biology, University of Hawai‘i at Mānoa, Kāne‘ohe, Hawai‘i, United States of America; Auburn University, UNITED STATES

## Abstract

A major challenge for coral reef conservation and management is understanding how a wide range of interacting human and natural drivers cumulatively impact and shape these ecosystems. Despite the importance of understanding these interactions, a methodological framework to synthesize spatially explicit data of such drivers is lacking. To fill this gap, we established a transferable data synthesis methodology to integrate spatial data on environmental and anthropogenic drivers of coral reefs, and applied this methodology to a case study location–the Main Hawaiian Islands (MHI). Environmental drivers were derived from time series (2002–2013) of climatological ranges and anomalies of remotely sensed sea surface temperature, chlorophyll-*a*, irradiance, and wave power. Anthropogenic drivers were characterized using empirically derived and modeled datasets of spatial fisheries catch, sedimentation, nutrient input, new development, habitat modification, and invasive species. Within our case study system, resulting driver maps showed high spatial heterogeneity across the MHI, with anthropogenic drivers generally greatest and most widespread on O‘ahu, where 70% of the state’s population resides, while sedimentation and nutrients were dominant in less populated islands. Together, the spatial integration of environmental and anthropogenic driver data described here provides a first-ever synthetic approach to visualize how the drivers of coral reef state vary in space and demonstrates a methodological framework for implementation of this approach in other regions of the world. By quantifying and synthesizing spatial drivers of change on coral reefs, we provide an avenue for further research to understand how drivers determine reef diversity and resilience, which can ultimately inform policies to protect coral reefs.

## Introduction

Understanding the drivers that cause changes in coral reef ecosystems is essential to designing management interventions that enhance positive outcomes and minimize negative impacts. While coral reef ecosystem structure and function vary naturally due to changes in environmental drivers [1–2], anthropogenic drivers are increasingly becoming the primary structuring forces of coral reef condition [3–5]. Many of these anthropogenic drivers have the potential to not only influence individual populations or ecological processes, but can also erode coral reef ecosystem resilience [6–10]. Accumulated evidence shows that coral reefs can shift from coral dominated to other undesirable alternative ecosystem states as a result of chronic human impacts [11–15]. Such reorganization of ecosystem structure and function may be difficult to reverse and may lead to loss of ecosystem services [16–19].

Disentangling the interacting effects of environmental and anthropogenic drivers across space and time requires coordinated quantification of a broad array of data over large spatio-temporal scales and the adoption of a macro-ecological approach to their analysis. In the absence of such comprehensive datasets, past efforts have been highly skewed towards environmental drivers, while anthropogenic impacts often are quantified by coarse proxies, such as human population density [20]. Such proxies often confound and conflate the effect of interacting individual drivers [15,4,21–22], and provide little predictive power at the relevant scales that decision-makers require to make difficult choices about how to apply limited resources to reduce local threats to coral reef health in the face of a rapidly changing ocean [23–24]. Emerging technology and data streams (e.g., global observing systems, citizen-science, and shared data repositories) increasingly allow for compiling and analyzing large data sets on both anthropogenic and environmental drivers over a broad range of scales, offering an unprecedented opportunity to study and understand ecosystem dynamics and swiftly inform management decisions. Big data refers broadly to the integration and communication of information in novel ways to produce valuable scientific insights about the world [25–26]. Such big data approaches have been harnessed to map drivers of ecosystem change and quantify spatial and temporal changes in cumulative impacts on the oceans at a global scale [5,27], and are increasingly being used to synthesize spatial data to determine drivers of coral reef ecosystem state [28–29]. Building on these past efforts, the overall goal of this study was to build a methodological approach to guide the synthesis and mapping of large spatio-temporal data sets, addressing critical issues of scale, data interoperability, and management relevance.

Ensuring actionable science and increased management uptake of scientific findings requires a comprehensive methodological framework for spatial data synthesis that engages managers from the initial phase in the driver identification, synthesis, and distillation process [30]. This study advances a methodological approach for guiding the synthesis and mapping of large spatio-temporal data sets to support coral reef ecosystem studies and management decision-making. Our first objective was to establish a methodological approach to support the spatial integration of data to map drivers on coral reef ecosystems. The methodological framework developed in this study involved four main steps: 1) development of a driver typology and approach for identifying management end-user needs, 2) establishment of the temporal and spatial scale(s) of analyses, 3) quantification and mapping of drivers, and 4) distilling and communicating driver data sets to managers and policymakers. Our second objective was to apply this methodological approach in a case study by quantifying and mapping environmental and anthropogenic drivers of coral reef ecosystem states in Hawai‘i. The case study site was chosen due to the geographic variability of impact gradients and coral reef ecosystem shifts documented on Hawaiian coral reefs [31–34]. This case study allowed us to establish and operationalize a methodological approach to spatially integrate data sets on coral reefs that can be applied to future studies in order to inform pressing problems facing coral reefs worldwide.

## Materials and methods

### Study area

The main Hawaiian Islands (MHI) consist of eight high volcanic islands. The archipelago’s isolation in the middle of the Pacific Ocean exposes reefs there to large open ocean swells and strong trade winds, which strongly influence the structure of the coral reefs. These dynamic natural processes and extreme isolation have sculpted distinctive marine communities, with 25% endemism, that play a valuable role as a global biodiversity resource [35–38]. Some of these endemics are dominant components of the coral reef community with extremely high conservation value [39–40]. In Hawai‘i, coral reef ecosystems play an important role in the culture, lifestyle, and economy, providing nearly $360 million annually in benefits to society [41,31].

**Fig 1:**
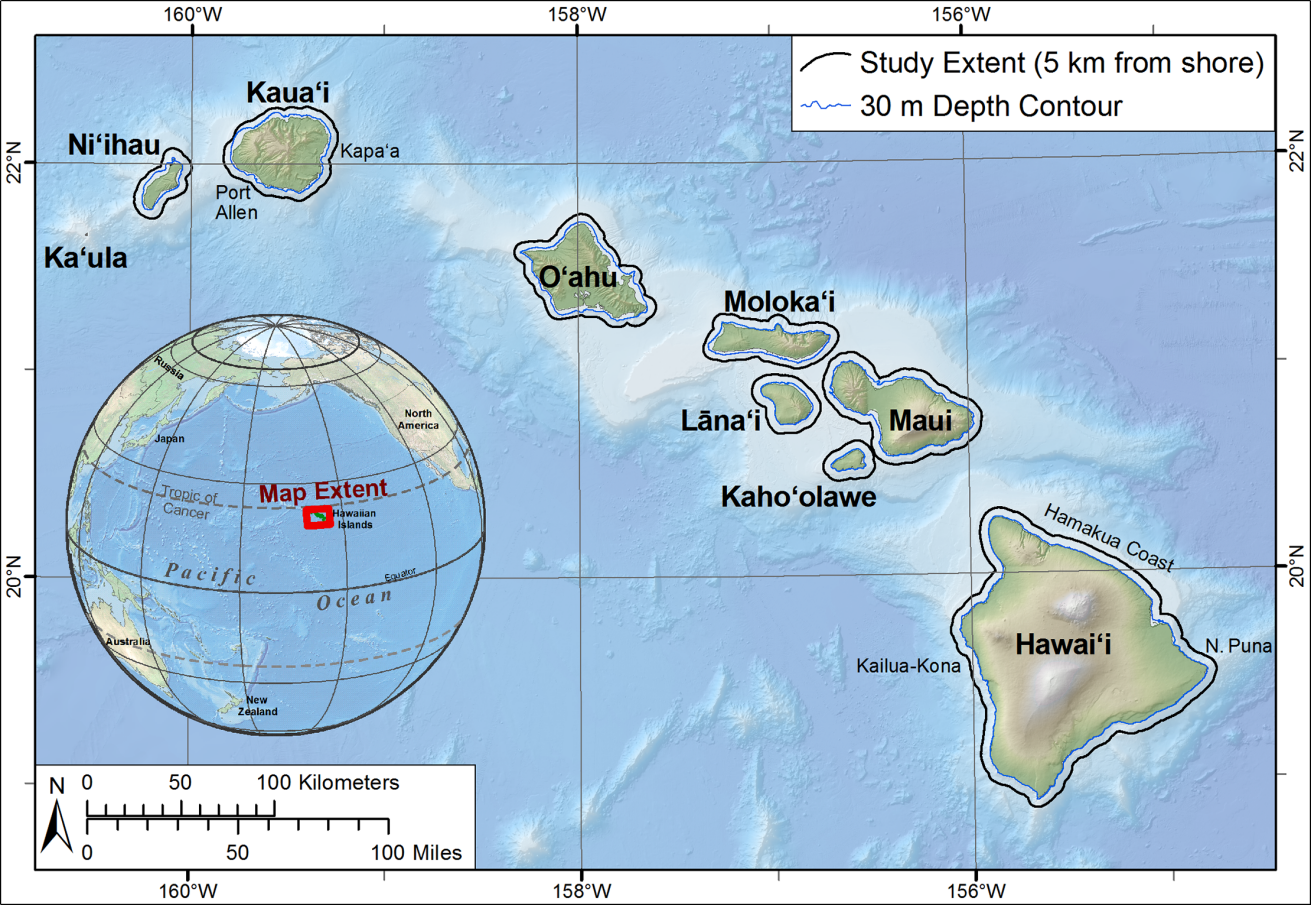
Study area. Map highlighting the main Hawaiian Islands study area and spatial footprint of anthropogenic and environmental driver data developed for this study extending offshore to 5 km. Biological monitoring data on coral reefs is generally shallower than the 30 m depth contour.

The study area encompassed all nearshore waters of the MHI from shore to 5 km offshore (Fig 1). Coral reefs have been in decline in Hawai‘i over the past 100 years due to the intense human pressure from a variety of overlapping uses such as recreational and commercial fishing, developed shorelines and watersheds, expanding ranges of invasive species, pollution, and other effects of an immense and growing coastal population and tourism industry [42,32,43]. The study area was chosen as a case study system because it contains gradients of environmental drivers, encompasses a broad range of human activities related to coral reefs, and prior to this study lacked sufficient synthesized data at spatial and temporal scales necessary to support ecosystem-based management of Hawaiian coral reefs.

### Methodological framework for spatial data integration

**Fig 2:**
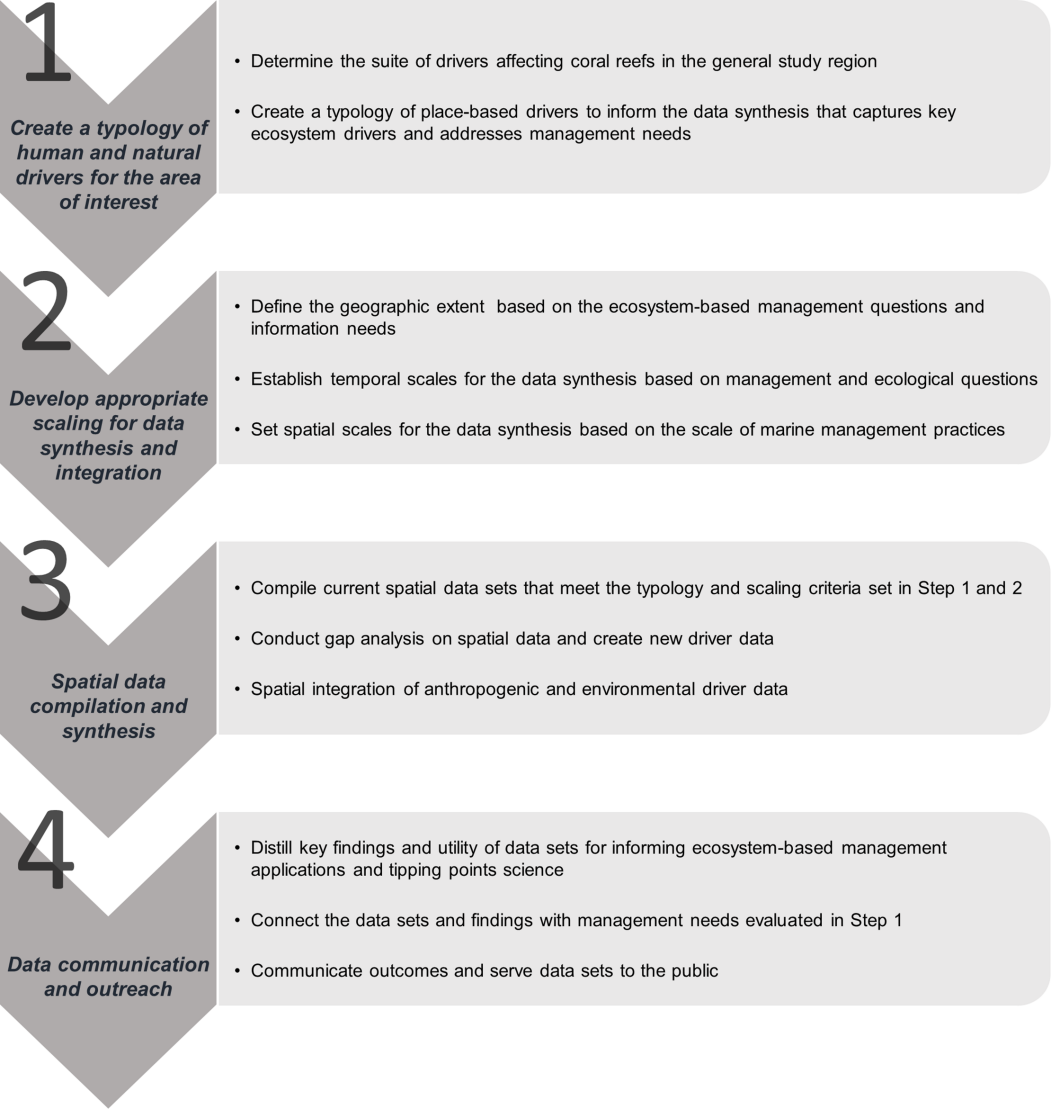
Methodological framework. Overall approach and steps to support the integration of spatial data to map human and natural drivers on coral reefs.

**Fig 3:**
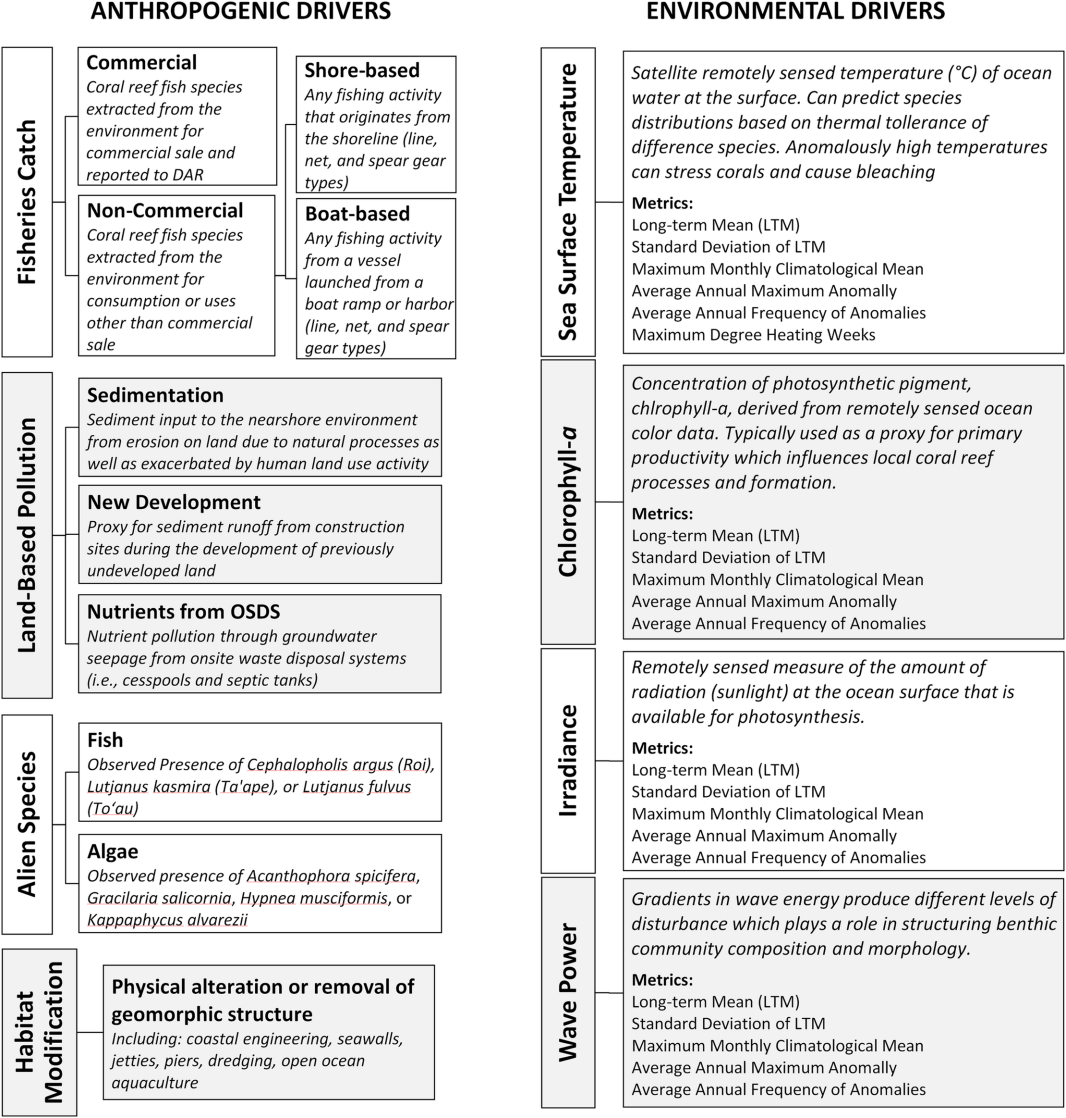
Anthropogenic and environmental drivers. Typology for primary proximate anthropogenic and environmental drivers for coastal waters of the main Hawaiian Islands from the shoreline extending 5 km offshore.

The methodological framework developed in this study involved four main steps to support the spatial data integration to map drivers on coral reefs across space and time (Fig 2). Our aim was to tackle the challenges of quantifying human uses and influences that have been poorly measured and/or difficult to access at fine spatial scales in the past. We synthesized numerous existing spatial data sets related to anthropogenic drivers and filled data gaps by developing models and specialized proxies to represent specific anthropogenic drivers, including fisheries catch from commercial and non-commercial fisheries (line, net, and spear gear types), land-based stressors (nutrients, sedimentation, new development), invasive species (fish and algae), and habitat modification. The typology of drivers created in step 1 was based on the key anthropogenic and environmental drivers identified by scientists, managers and key stakeholders in the study area (Fig 3). For instance, managers from the NOAA Office of National Marine Sanctuaries and State of Hawai‘i Division of Aquatic Resources (DAR) were engaged before the research started in order to provide input on the framing of the project and identify key drivers to include in the typology that they estimated to be most impactful on coral reefs. The typology expanded on a social-ecological framework that identified the primary human impacts that mediate the condition of coral reef ecosystems by Kittinger et al. [44] and integrated key environmental drivers that were identified by Gove et al. [45]. Specifically, we included environmental forcings known to be major drivers of coral reef ecosystem state, namely sea surface temperature (SST), chlorophyll-*a* (a proxy for phytoplankton biomass), irradiance, and wave power [4].

The second step involved the development of appropriate scaling (geographic extent, temporal, and spatial resolution) to inform the data synthesis based on coral reef management information needs at the state level (Table 1). In Hawai‘i, as in many coral reefs globally, the ocean is managed at multiple scales (state-wide, regionally, and locally). Accordingly, the scale and geographic extent of the data synthesis and integration were guided by the planned management application and utility of these data within the constraints of native spatial resolutions of input data. The spatial scale in relation to grain size (pixel size) of the data sets were defined by each data source. The finest grain size was created for each data source in order to provide managers with the highest resolution data possible. Recommendations from the managers guided the geographic extent of the data. For a majority of the data sets, the temporal scale represented approximately a ten-year average, which provided managers with a data set that gave a broader temporal understanding of drivers across space instead of a single snapshot in time. Our goal was to create driver data sets at the finest spatial scale possible that supports management information needs and this step was carried out with federal and state-level coral reef managers.

**Table 1:** Anthropogenic and environmental drivers mapped and input data sources.

**Anthropogenic Drivers**	**Units**	**Spatial resolution**	**Temporal range**	**Data Source**
Fisheries Catch—Commercial	annual average catch in kg/ha by gear (line, net, and spear)	100 m	2003–2013	Reported Commercial Catch 2003–2013 (DAR); Commercial Reporting Blocks (OP)
Fisheries Catch -Non-Commercial (Shore-Based)	annual average catch in kg/ha by gear (line, net, and spear)	100 m	2004–2013	Island-scale estimates of catch (kg/yr by gear) from MRIP 2004–2013 [50]; USGS DEM (slope); TIGER Roads; HMRG Bathymetry Synthesis
Fisheries Catch -Non-Commercial (Boat-Based)	annual average catch in kg/ha by gear (line, net, and spear)	100 m	2004–2013	Island-scale estimates of catch (kg/yr by gear) from MRIP 2004–2013 [50]; Boating Facility locations (OP); Human population (US Census, 2010)
Sedimentation	average annual amount of sediment (tons/yr)	100 m	2005	InVEST Sediment Delivery Ratio Model output [57]; National Hydrography Dataset
New Development	relative level of new development	100 m	2005–2011	NOAA C-CAP 2005-2010/11; NHD Watersheds; Distance from shore
Nutrients from OSDS	g/day and effluent in gallons/day	500 m	2009–2014	OSDS Point location and estimated effluent/nutrient flux [61–62]
Invasive Species	Presence only of invasive fish and algae	500 m	2000–2013	Hawaii Monitoring and Research Collaborative Database (2000–2013); Invasive marine algae surveys [64]
Habitat Modification	presence of habitat modifying features	500 m	2001–2013	NOAA CCMA Habitat Maps (2007); NOAA ESI lines (2001); Maintained Channels (2013); Offshore Aquaculture point locations
**Oceanographic Drivers**	**Units**	**Spatial resolution**	**Temporal range**	**Data Source**
Sea Surface Temperature	° Celsius	5 km	2000–2013	NOAA Pathfinder, NOAA/NESDIS/STAR Blended SST 0.1 and 0.05 degree (weekly composites)
Chlorophyll-a	mg/m^3^	4 km	2002–2013	MODIS (8-day composites)
Irradiance	Einstein m-^2^ d^-1^	4 km	2002–2013	MODIS (8-day composites)
Wave Power	KW/m	0.5–1 km	2000–2013	Simulating Waves Nearshore (SWAN) model–(hourly)

Input data sets used to develop continuous spatial layers of anthropogenic and environmental drivers for coastal waters of the Main Hawaiian Islands from the shoreline extending 5 km offshore. The following climatological metrics were calculated for each environmental driver: long-term mean, standard deviation of long-term mean, climatological maximum, average annual maximum anomaly, frequency of anomalies, and for SST only: maximum degree heating weeks. Acronyms: DAR–Hawai‘i Division of Aquatic Resources; OP—Hawai‘i Office of Planning; MRIP—Marine Recreational Information Program; USGS—United States Geological Survey; DEM—Digital Elevation Model; InVEST—Integrated Valuation of Ecosystem Services and Tradeoffs; NHD—National Hydrography Dataset; OSDS—On Site waste Disposal Systems; NOAA—National Oceanic and Atmospheric Administration; CCMA—Center for Coastal Monitoring and Assessment; ESI—Environmental Sensitivity Index; C-CAP—Coastal Change Analysis Program; TIGER–Topologically Integrated Geographic Encoding and Referencing; HMRG–Hawai‘i Mapping Research Group; NESDIS STAR–National Environmental Satellite, Data, and Information Service, Center for Satellite Applications and Research; SST–Sea Surface Temperature; MODIS–Moderate Resolution Imaging Spectroradiometer

The third step involved the quantification of anthropogenic and environmental drivers that are known from the literature to be major drivers of coral reef ecosystem state. We extended the island-scale modeled and satellite-based metrics of environmental drivers developed by Gove et al. [45] to intra-island spatial scales. In addition, we created maps of anthropogenic drivers that have been poorly measured and/or difficult to access at this scale in the past (e.g., fisheries catch, sedimentation, nutrients) and synthesized current data sets on habitat modification and invasive species. During this step, the project team met with state and federal management staff to receive feedback on the proposed methodology and to vet the driver data sets. The final key step in the process involved distilling, communicating, and serving these datasets to ensure their use in future research, so as to broaden our understanding of the coral reef ecosystem state and inform best ecosystem-based management practices.

### Anthropogenic driver data and spatial analysis

#### Fisheries catch

Nearshore wild-capture food fisheries in Hawai‘i consist of diverse groups of fishers using a wide array of gears and targeting hundreds of species [46–48]. Overfishing can cause ecosystem degradation and long-term economic loss. Fine-scale information on catch and effort for Hawaiian nearshore fisheries is virtually nonexistent. Commercial catch of reef fishes is reported to the State of Hawai‘i DAR based on large reporting blocks; furthermore, it constitutes a very small proportion of all reef fishes caught and is unrepresentative of nearshore fisheries as a whole [49–50]. McCoy [50] examined 10 years of data from the NOAA Marine Recreational Information Program (MRIP) and other data sources, including creel (angler) survey data, to estimate non-commercial nearshore catch by gear type and platform (boat- or shore-based) for each island and found this catch to be, on average, 10 times greater than the reported commercial catch.

To map the average annual catch of reef fishes for the MHI, we combined commercial reported catch with island-level estimates of non-commercial catch from McCoy [50], along with an index of accessibility for shore- and boat-based fishing. We created nine separate data layers for fishing, grouped into three categories: 1) commercial fishing, 2) non-commercial shore-based fishing, and 3) non-commercial boat-based fishing, each with three gear classes: line, net, and spear fishing. We excluded invertebrates and coastal pelagic finfishes (e.g., *Selar crumophthalmus* and *Decapterus* spp.) from the fisheries data layers since these taxa were either loosely reef-associated or were poorly represented in the biological datasets. Each final data layer represented an annual average catch in kg/ha, at 100 m (1 ha) spatial resolution.

For ***commercial fishing*,** we calculated the average annual catch of reef fishes by gear category (line, net, and spear) over the years 2003–2013 as reported in commercial catch data by large irregular reporting blocks (50–250 km^2^), collected by State of Hawai‘i DAR Commercial Marine Landings Database (CML). The gear types reported in the CML database do not distinguish boat- from shore-based gears.

For ***non-commercial fishing*,** we used estimates from McCoy [50] of average annual catch by platform (boat, shore) and gear type at the island scale, from 2004–2013 derived from MRIP combined fisher intercept and phone survey data [50]. To spatially distribute these island-scale estimates of catch offshore around each island, we developed and used spatial proxies for accessibility to fishers. For ***shore-based non-commercial fishing***, we combined two different measures of shoreline accessibility (terrain steepness and presence of roads) to define a total of nine accessibility categories which were then weighted with respect to fisheries catch based on expert opinion (see S2 Supplement for detailed methodology and weighting factors). For ***boat-based non-commercial fishing***, we combined over-water distance to boat harbors and launch ramps with a Gaussian decay function that assumed the majority of catch occurs within 15–20 km of each harbor, and weighted the amount of catch out of each ramp/harbor by the human population within 30 km of the harbor or ramp (See S2 Supplement for details). Spatial analyses were run separately for each boat harbor or launch ramp (so that footprints from nearby harbors could overlap), and then catch surfaces for each harbor/ramp were summed. The decay functions, distances, and weighting factors used to derive non-commercial fishing layers were vetted with resource experts and managers in absence of empirically based values.

For each of the 9 fishing layers described above, marine protected area boundaries and military restricted areas were used to adjust catch according to specific restrictions in each area. We conducted a comprehensive review of existing state and federal regulations in order to update military restricted areas and outdated marine managed area boundary data for the State of Hawai‘i, and then evaluated each area with regard to the 9 fishing categories mapped. Fish catch incomplete no-take MPAs was set to zero and reduced in other areas with restricted access according to expert input and local knowledge. The final units for each layer were converted to average annual catch in kg/ha, so that the scale was consistent across layers and could be summed across different combinations of gear types and platforms (e.g., total spearfishing catch across all platforms, or total catch from the non-commercial sector only).

Non-commercial fishing maps were validated using estimates of annual catch of reef fishes compiled from existing site-based intercept surveys from creel survey reports (see S1 Supplement for creel survey references). Creel surveys estimate catch and effort in a particular area using a sampling program that involves fisher interviews and inspection of catch. Survey areas described or mapped in creel survey reports were digitized and used to sum catch for the corresponding areas from the total non-commercial catch map. A least-squares linear regression with intercept anchored at the origin was performed to compare the two sets of estimates and compared to the 1:1 line (see S1 Supplement).

#### Land-based stressors

Sediment from various land-based stressors can affect reef health by smothering corals and blocking light, leading to degradation of reef ecosystems [51]. In addition, excess nutrients can trigger macroalgal blooms that smother and kill corals [52–53]. In order to map land-based stressors, we developed tailored models of nutrient and sediment impacts to reefs by combining estimates of loads with ecologically informed models of their spatial distribution into the nearshore. To quantify sedimentation, we used the Integrated Valuation of Ecosystem Services and Tradeoffs (InVEST) sediment delivery ratio model [54–55] to estimate sediment delivery for each of the eight MHI [56]. The model was customized and parameterized for Hawai‘i, and calibrated using scientific data on sediment loads [57]. The model predicted average annual sediment export (tons/yr) from each terrestrial map pixel as a function of vegetation cover characteristics, geologic substrate, soil erodibility, rainfall erosivity, and slope. The resulting modeled sediment loads were aggregated by drainage basin to each point where a stream meets the coast and then dispersed offshore using the Kernel Density tool in ArcGIS, resulting in a map of sediment plumes 1.5 km offshore with 100 m cell size. The Density tool was run iteratively for each pour point, land area was clipped out, and offshore values were back-calculated to sum to the input sediment load. Finally, each individual plume raster was added together with Cell Statistics (See S1 Supplement for details).

The InVEST Sediment Delivery Ratio model was based on static land use land cover data and as a result, sources of sediment not captured in this model included new construction sites that strip land of vegetation, leaving bare soil s vulnerable to erosion, and often harbor additional large piles of soil on site for grading and landscaping. To capture this source of land-based pollution, we identified areas that had been newly developed over a recent five-year period using high resolution data from NOAA’s C-CAP (Coastal Change Analysis Program). We identified all map pixels that changed from undeveloped land to a hard, man-made surface from 2005 to 2010, and calculated the area of new development per watershed. We used a Gaussian function that decays with distance from shore (similar to that used by the ArcGIS Kernel Density tool for the sediment layer) to disperse these watershed-scale values offshore and approximate the dispersal of sediment from new construction into the nearshore environment. Values were re-scaled from 0 to 1 in order to represent the relative level of new development with the final layer having 100 m cell sizes.

Hawai‘i has the highest number of onsite waste disposal systems (OSDS) (i.e. cesspools and septic tanks) per capita in the U.S., many of which are adjacent to the coastline (EPA—https://www.epa.gov/uic/cesspools-hawaii). These OSDS leach excess nutrients and pollutants into groundwater that flows to the ocean [58]. Excess nutrients can promote rapid algal growth, outcompeting corals and disrupting the ecosystem [59–60]. To represent this impact spatially, we used data on OSDS in the form of point data from the University of Hawai‘i and Hawai‘i Department of Health [61–62]. Data consisted of estimated nitrogen flux and phosphorous flux from each Tax Map Key parcel with OSDS in units of kg/day and effluent in gallons (3.8 L) per day. We converted the points to a raster by summing nutrient flux values within 500 m x 500 m pixels. Focal statistics were used to calculate the total flux within a 1.5 km radius of each cell based on sediment plume extents measured by Ostrander et al. [63]. We produced three final layers at a cell size of 500 m: nitrogen, phosphorus, and total effluent flux within 1.5 km of each map pixel with final units of g/day/7 km^2^ for nutrients and gal/day/7 km^2^ for effluent (7 km^2^ ≈ area of a circle with 1.5 km radius).

#### Invasive species

Several species of alien algae have become invasive in Hawai‘i (e.g., *Acanthophora spicifera*, *Gracilaria salicornia*) [64]. Likewise, several fish species have become invasive following intentional introductions as food fish in the 1950s [65]. Two data layers were created to characterize the presence of invasive fishes and invasive algal species in nearshore waters of the MHI. The invasive algae data were from surveys conducted in 2002 and data from the Hawaii Monitoring and Research Collaborative, which synthesized underwater visual surveys from multiple sources on fishes and benthic assemblages over the years 2000–2013 [66–67]. Transects were categorized with presence of invasive fish species (*Cephalopholis argus*, *Lutjanus kasmira*, *Lutjanus fulvus*) and invasive algae species (*Acanthophora spicifera*, *Gracilaria salicornia*, *Hypnea musciformis*, *Kappaphycus alvarezii*) and these point data were converted to raster. To account for uncertainty in geographic position, and movement of the fish species or fragmentation and spread of algae, focal statistics were run to calculate presence within a 1 km radius of invasive algae observations and a 2 km radius of invasive fish observations. A 2 km buffer was used for fishes based on literature about the home ranges of *C*. *argus* and *L*. *kasmira* [68–69] and a 1 km scale was used for invasive algae based on Smith et al. [70]. The layers represent the presence only of invasive fishes and algae, with a cell size of 500 m.

#### Habitat modification

Coastal habitats are under increasing pressure and use from anthropogenic activities. Here we defined habitat modification as the alteration, or removal of geomorphic structure, as a result of human use. We mapped the presence of habitat modifying features like seawalls, piers, breakwaters, dredged areas, artificial land (i.e. filled wetlands), and offshore structures by combining several existing datasets derived primarily from satellite and aerial imagery. We integrated the following data sets into the habitat modification layer: 1) artificial shoreline, 2) maintained channels and dredged areas, and 3) offshore aquaculture. The layer represents the presence or absence of habitat modification, with a cell size of 500 m.

### Environmental driver data and spatial analysis

Proper characterization of environmental drivers through space and time is critical to understanding the intrinsic biophysical interactions occurring within coral reef ecosystems. Here, we built upon previous work by Gove et al. [45] and developed a suite of metrics for four environmental drivers of coral reefs (sea surface temperature, Chlorophyll- *a*, irradiance, and wave power) at 0.5–4 km spatial resolution across the MHI.

Sea surface temperature (SST) plays an important role in a number of ecological processes occurring within coral reef environments and can vary in response to diel, intra-seasonal (e.g. mesoscale eddies), seasonal, inter-annual (e.g. El Nino Southern Oscillation) and decadal (e.g. Pacific Decadal Oscillation) forcing. SST (°C) was quantified weekly at 5 km from multiple satellite-derived data sets, including NOAA’s Pathfinder v5.2 and NOAA’s Center for Satellite Applications and Research blended 11 km and 5 km daily data set, available from 1985–2013. A bias adjustment was applied, derived from linear regression to the overlap periods of datasets. Data were excluded if deemed of poor quality (quality value < 4, [71]) or if individual pixels were masked as land (see S1 Supplement for more detail).

Chlorophyll-*a* is a widely used proxy for phytoplankton biomass (e.g., Gove et al. [72]) and as an indicator for changes in phytoplankton production (e.g., Chassot et al. [73]), an essential source of energy in the marine environment [74]. Irradiance represents the amount of solar radiation (sunlight) at the ocean surface that is available for photosynthesis. Chlorophyll-*a* (mg/m^3^) and irradiance (Einstein/m^2^/d) were obtained from NASA’s 4 km, 8 day, Moderate Resolution Imaging Spectroradiometer (MODIS; http://oceancolor.gsfc.nasa.gov/cms/) data set available from July 2002 to present. Following Gove et al. [45], a multistep masking routine was applied to remove spurious data associated with optically shallow waters (< 30 m) and errors induced by terrigenous input, re-suspended material, or bottom substrate properties [75] (see S1 Supplement for more detail).

Gradients in wave forcing result in varying levels of disturbance underwater that have strong implications for both benthic and fish communities in coral reefs [2,76]. Wave power (kW/m), which incorporates both wave period and wave height and therefore represents a more realistic estimate of wave-induced stress on coral reefs, was obtained using University of Hawai‘i SWAN (Simulating WAves Nearshore) wave model, available at 1 hr, 0.5 km resolution from 1979–2013 [77]. Daily maximum wave power was calculated from the hourly data set. Spatial mismatch between model resolution and the high degree of wave refraction, amplification, and dissipation resulted in spurious wave power values in close proximity to shore. As such, all model pixels adjacent to shore (≤500 m) were removed prior to analysis (see S1 Supplement for more detail).

We quantified a suite of metrics in order to effectively capture the ecological relevance of each environmental driver. Monthly climatologies were first calculated utilizing the full time range of data availability. The maximum monthly mean, or the largest value of the 12 monthly climatological values, was selected to represent the upper limit in the ‘normal’ range of environmental conditions [78–79]. Over time, coral reefs have adapted to exist within a particular climatological range; an envelope of environmental forcings that is region-specific and governed by a reef’s geographic location [45]. Anomalous events were then calculated for environmental conditions that exceeded the maximum monthly mean. Specifically, the annual average in the total number and magnitude of anomalous events were quantified for each environmental driver. The long-term mean and standard deviation were also quantified to capture average environmental conditions and the associated time-dependency in those conditions. Finally, the maximum Degree Heating Weeks (DHW; °C-weeks; www.coralreefwatch.noaa.gov), calculated from SST, was also included as a metric of thermal stress on corals [80–81]. Anomaly, climatological maximum, long-term mean, standard deviation and DHW metrics were calculated from 2000–2013. Owing to data availability limitations, metrics for Chlorophyll-*a* and Irradiance were calculated from July 2002 –December 2013. For all environmental drivers and metrics, nearshore map pixels with no data were filled with values from the nearest neighboring offshore pixel.

### Driver correlations across islands

We carried out a principle components analysis (PCA) at the island scale to determine which islands share similar sets of dominant drivers in order to help managers gain understanding on the varying needs and priorities for each island based on the extent of dominant drivers. To compare general patterns of anthropogenic and environmental drivers across islands, summary statistics were derived to calculate an island mean for each variable. For each island, a raster mask was created and values within the mask were used to calculate minimum, lower quartile, median, upper quartile, and the maximum, and displayed as boxplots. Median values were then used in a principle components analysis to evaluate correlations among variables across islands. For environmental drivers, only the climatological maximum metrics were included in the PCA.

## Results

### Spatial patterns of anthropogenic drivers–the Hawaiian Islands as a case study system

#### Fisheries catch

The greatest mapped values of commercial and non-commercial boat-based reef fish catch occurred on the island of O‘ahu, with commercial catch highest off west O‘ahu and the highest non-commercial boat-based catch near Honolulu on the south shore. Across the MHI, catch by the line gear type was at least two times greater than spear or net for non-commercial shore-based fishing, and as much as 10 times greater in the case of shore-based net fishing on Hawai‘i Island. Maui had the highest catch per unit area for shore-based net fishing. Pockets of highly accessible coastline on Hawai‘i Island had the highest total combined catch per unit area in the state (max. value of 40.4 kg/ha on Hawai‘i compared to 29.2 kg/ha on O‘ahu). However, Hawai‘i Island also had large expanses of inaccessible and relatively unfished coastline compared to O‘ahu, which has more reef area and nearly all shorelines are highly accessible, resulting in catch being more widely dispersed. Other areas with exceptionally low catch included the islands of Ni‘ihau and Kaho‘olawe.

**Fig 4:**
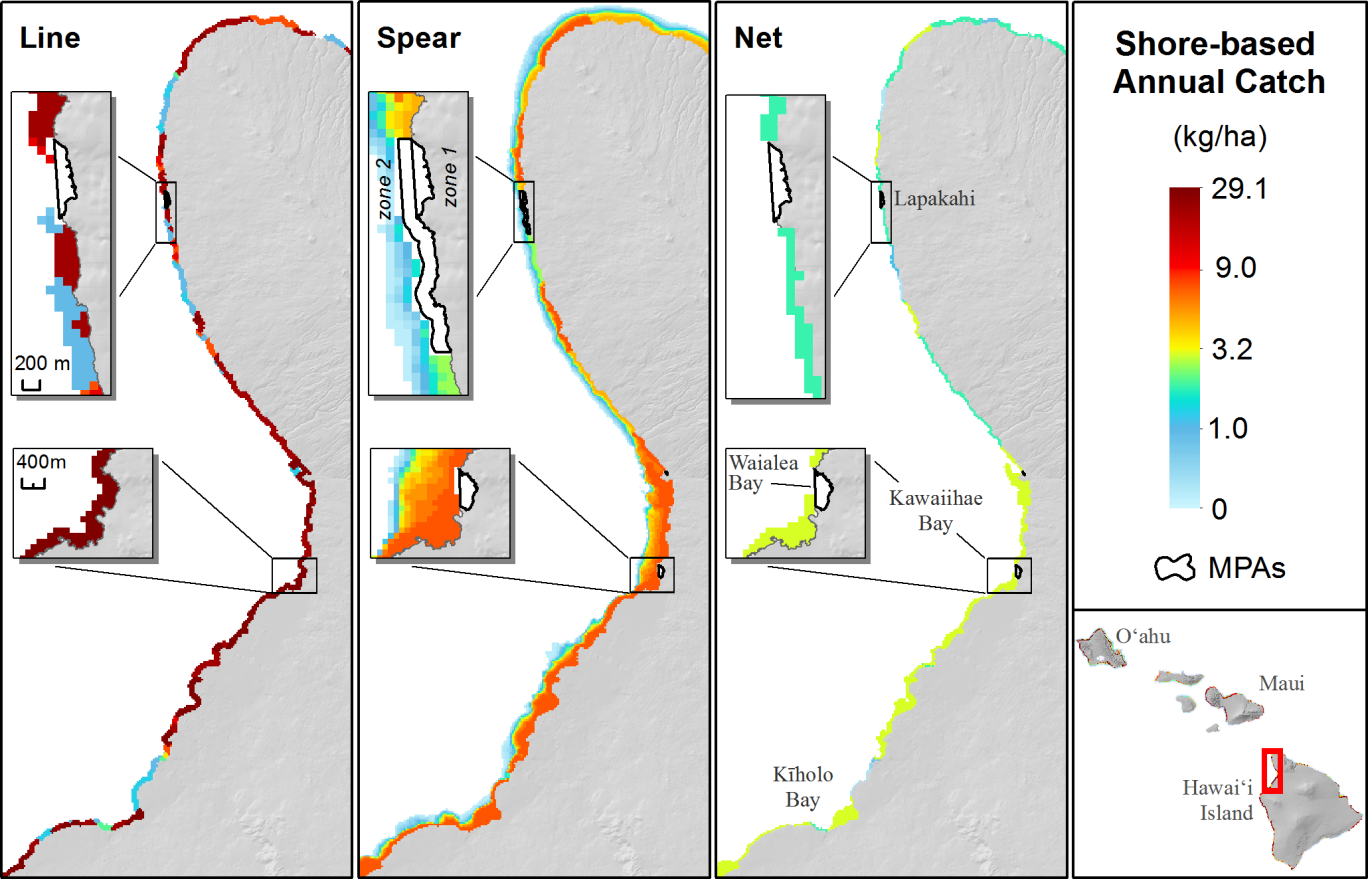
Non-commercial shore-based fishing. Maps of the final continuous spatial layers for non-commercial shore-based fishing catch (kg/ha) on the Kohala coast of the Island of Hawai‘i. Maps depict the average annual catch of reef fish by non-commercial shore-based fishing with line, spear, and net gears (left to right, respectively). Inset maps on each panel show examples of Marine Protected Areas (MPAs) with different gear restrictions. Only MPAs that completely prohibit use of the respective gears are shown on each panel. Upper inset = Lapakahi Marine Life Conservation District (MLCD): zone 1 is full no take, zone 2 allows line and net fishing but prohibits spearfishing. Lower inset = Waialea Bay MLCD: line fishing is allowed but spear and net are prohibited.

On the Kohala Coast of Hawai‘i Island, shore-based non-commercial fishing pressure was relatively high from Kawaihae Bay south to Kīholo Bay, but was more variable and demonstrated localized hotspots of accessibility north of Kawaihae Bay (Fig 4). The inset panels in Fig 4 illustrate how marine protected areas (MPAs) with varying harvest control rules and restrictions on different gear types were accounted for. For example, the Lapakahi MPA has one zone that is fully no take, but allows line and net fishing in the outer zone. The Waialea Bay MPA allows shore-based line fishing throughout the area but prohibits spear or net. Fishery catch maps significantly predicted creel survey data (*p*<0.005, R^2^ = 0.64) and the fitted regression line (slope = 0.99) was close to 1:1 (Fig A in S1). In terms of absolute values of average annual catch, the largest differences between our maps and creel surveys results were in Kīholo Bay (which our maps underestimate by 2,700 kg compared to creel results), and waters of Waikiki outside of MPAs (which our maps overestimate by 2,100 kg).

#### Land-based stressors

**Fig 5:**
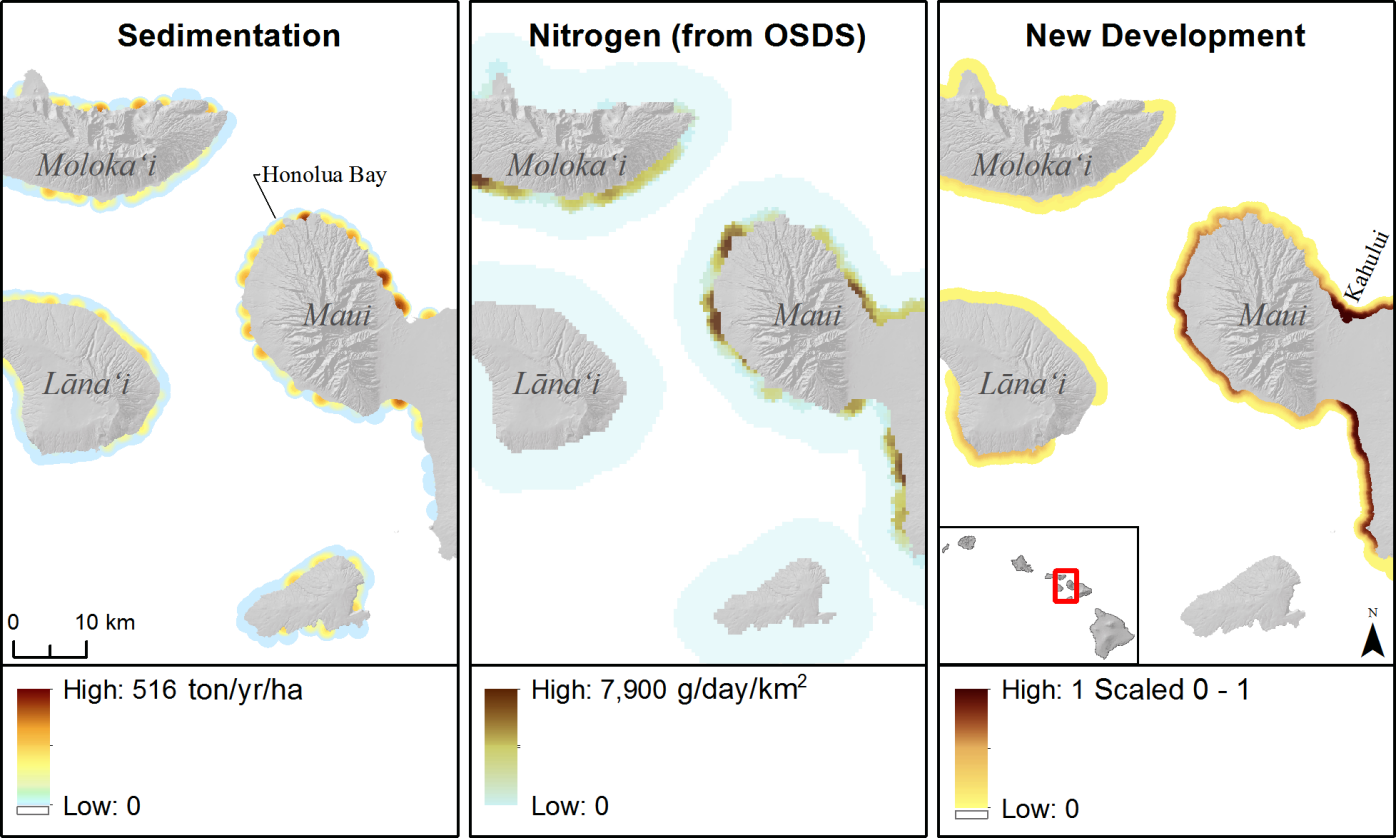
Land-based pollution. Maps of land-based pollution in central Maui Nui. From left to right: sedimentation (tons of sediment/yr/ha), nitrogen flux from onsite waste disposal systems (OSDS) (g/day/km^2^), and new development (scaled 0–1) which represents the impact of sediment runoff from recent construction sites on newly developed land between 2005–2011.

Mapped outputs of land-based stressors showed highly localized hot spots across the MHI with the greatest values for sedimentation and nutrients occurring in specific locations on Maui, Hawai‘i Island, and the North Shore of O‘ahu. For instance, the highest sediment load across the state occurred at Kaiaka Bay on the north shore of O‘ahu. At the fine-scale, we found that many enclosed embayments or shallow coastal locations with low wave energy were characterized by high levels of sedimentation and nutrients, and often high chlorophyll-*a* values. As an example, Honolua Bay, on the northwest coast of Maui, demonstrated these localized spatial patterns and had regionally high sediment loads for the West Maui watersheds (Fig 5).

New development and on site waste disposal (nutrients–nitrogen and phosphorus flux) had spatial patterns of overlap with the high sediment loads along the coastline surrounding Kaiaka Bay and Hale‘iwa on the north shore of O‘ahu. Across the MHI, the 50 largest single OSDS effluent loads occurred on Maui and Hawai‘i Island.The maximum estimated flux of nutrients into nearshore waters from onsite waste disposal effluent occurred in south Kailua-Kona town on Hawai‘i Island, Kaiaka Bay on O‘ahu, and Kapa‘a on Kaua‘i. Coastal watersheds with the highest amount of new development (i.e. area converted to impervious surfaces) included the south shore of O‘ahu, Kahului and much of central Maui (Fig 5), and the northern Puna district on Hawai‘i Island. Kaua‘i and Maui islands both had high mapped new development and high OSDS nutrient flux combined. For instance, both nutrients from on-site waste disposal and sedimentation were elevated in southwest Kaua‘i (e.g., Waimea River near Port Allen).

#### Invasive species

**Fig 6:**
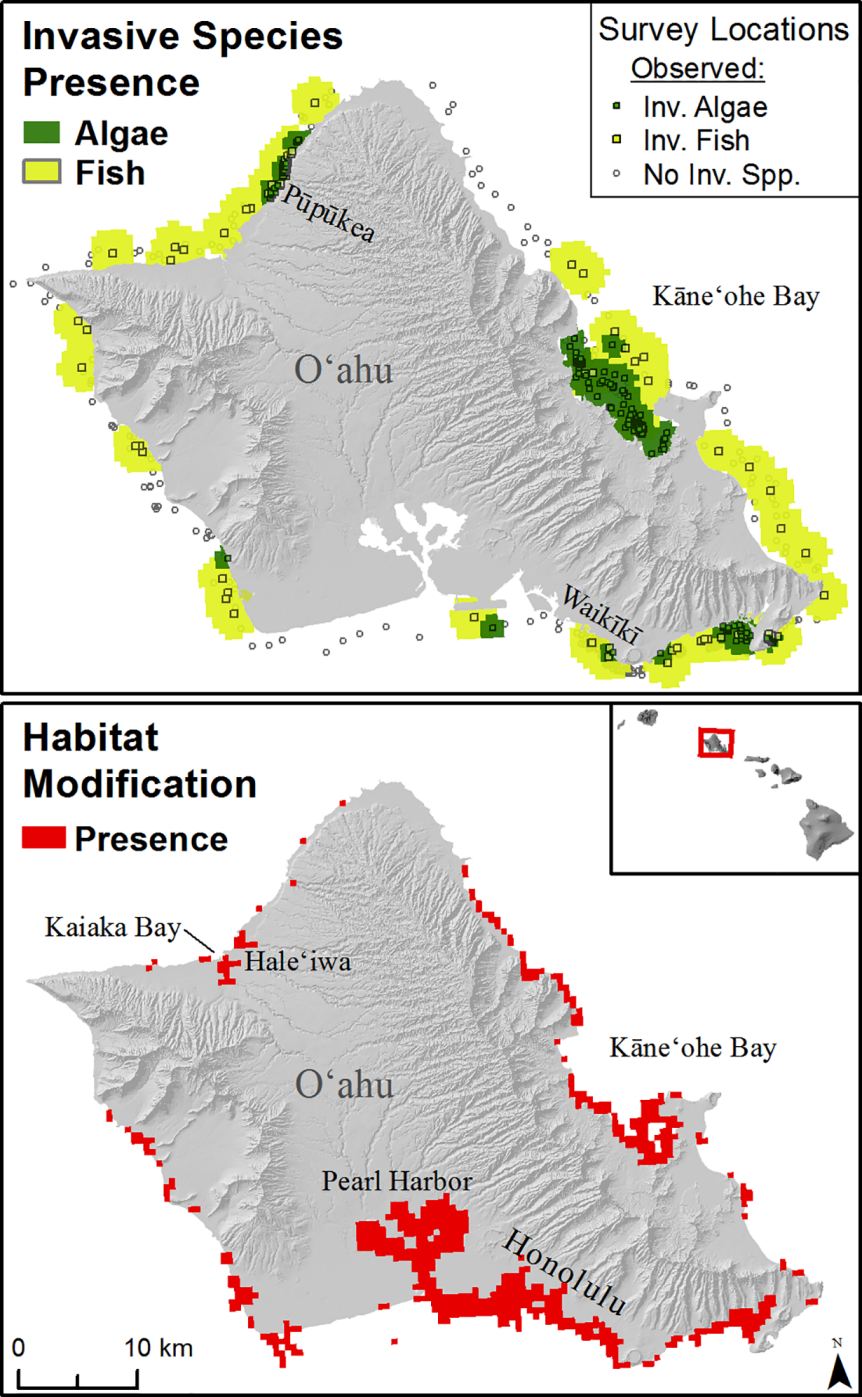
Invasive species and habitat modification. Top: Invasive species (presence only) on O‘ahu, Hawai‘i (green-invasive algae, yellow-invasive fish). Invasive algae layer is displayed on top of invasive fish. Bottom: Habitat modification (red) present on O‘ahu, Hawai‘i including manmade and artificial shorelines, maintained channels and dredged areas, and offshore aquaculture.

Invasive fish species were present on all islands. On O‘ahu, our maps show a majority of the nearshore to be invaded by non-native fishes (Fig 6). Invasive algae species are known to occur in certain discrete locations, particularly Kāne‘ohe Bay and Pūpūkea on O‘ahu (Fig 6), West Maui, south shore of Moloka‘i, and scattered around the other islands.

#### Habitat modification

Habitat modification was abundant at the most populated areas across the MHI such as O‘ahu, Hilo on Hawai‘i Island, and west Maui. The south shore of Moloka‘i also stands out due to numerous remnant native Hawaiian fishpond walls, as well as many dredge scars. The largest areas of continuous habitat modification were on O‘ahu from Waikīkī to Pearl Harbor, and Kāneʻohe Bay (Fig 6), which have extensively armored and developed shorelines, as well as the largest human populations in the state.

### Spatial patterns of environmental drivers

#### Wave forcing

**Fig 7:**
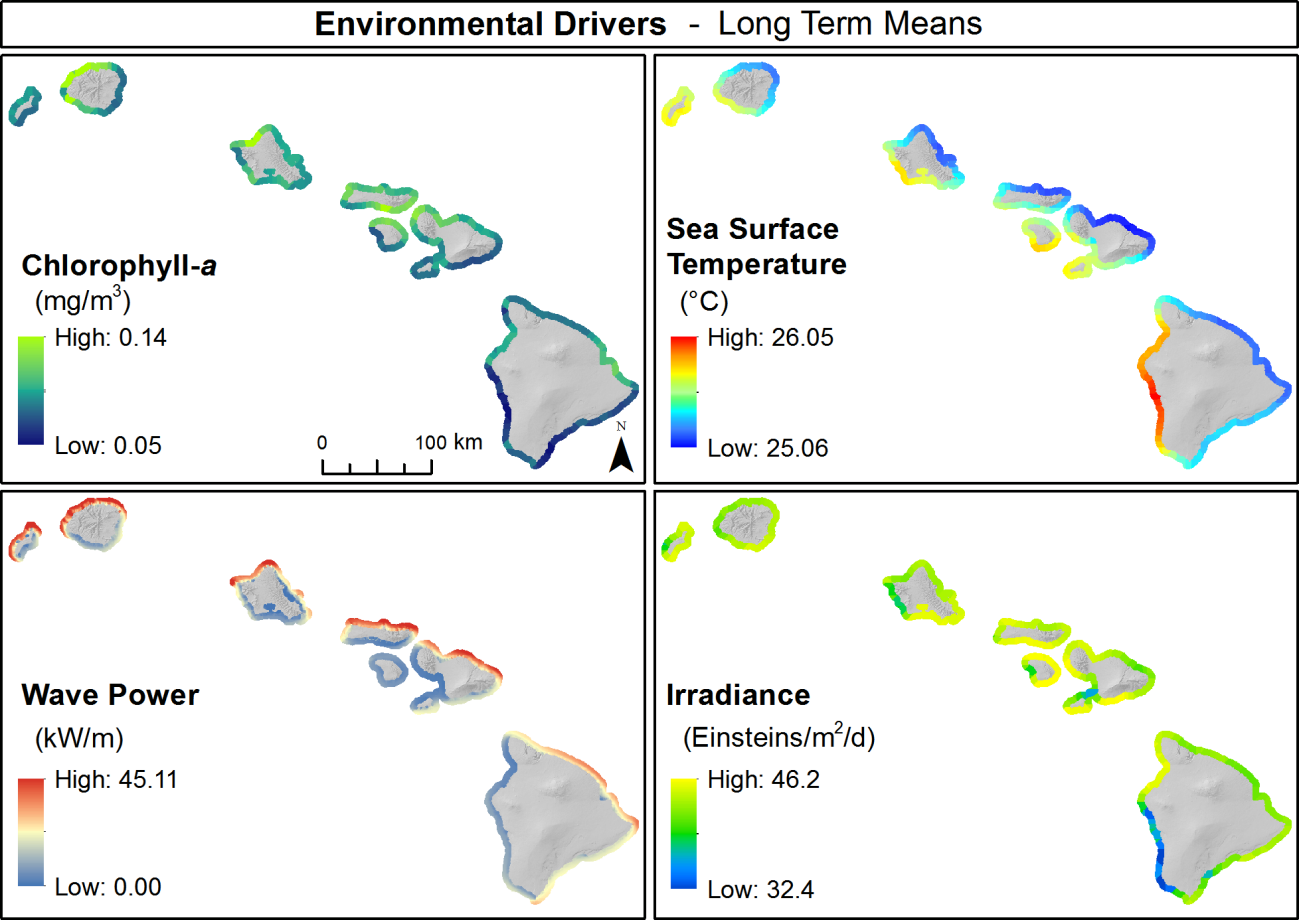
Environmental drivers. Spatial distributions in key environmental drivers that influence coral reef ecosystems, including chlorophyll-*a* (mg m^-3^), sea surface temperature (°C), wave power (kW m^-1^), and irradiance (Einstein m^-2^ d-^1^) across the eight main Hawaiian Islands.

Hawai‘i receives large ocean swell from storms in the northwest Pacific that predominantly impact the northwest facing shorelines of the MHI (Fig 7). However, because of the northwest to southeast orientation of the Archipelago, islands located further northwest (i.e. Ni‘ihau, Kaua‘i, O‘ahu) generally received greater levels of wave forcing and cause a blocking effect of ocean swells reaching islands located to the southeast, dramatically reducing the levels of wave energy hitting the coastlines of these islands. The blocking effect is readily seen in the long-term mean (Fig 7), climatological maximum and average annual maximum anomaly in wave forcing along the west coast of Lāna‘i, Maui, and Hawai‘i Island compared to that observed along the northwest coast of Ni‘ihau, Kaua‘i, and O‘ahu.

#### Sea surface temperature (SST)

SST exhibited a consistent spatial patterning across the MHI; windward facing coastlines of all islands had generally cooler ocean temperatures compared to leeward facing coastlines (Fig 7). The spatial pattern in SST was particularly amplified on Hawai‘i Island, where the west side of the island was dominated by warmer ocean temperatures compared to the east side. The MHI are exposed to trade winds that blow from the northeast for a majority of the year. These winds drive vertical mixing of the upper water column, bringing cooler ocean temperatures to the near surface. As such, easterly facing coastlines exposed to trade winds predominantly have cooler SSTs compared to coastlines that are more sheltered.

#### Irradiance

Spatial distribution in irradiance showed no clear and consistent patterning across the MHI. Irradiance values were greatest along the southern coasts of O‘ahu and Moloka‘i, northeasterly coast of Lāna‘i, and southwest coast of Maui (Fig 7). The southern half of the west coast of Hawai‘i Island had the lowest long-term mean and climatological maximum irradiance values, but also had the greatest maximum anomaly values of any island.

#### Chlorophyll-a

Across the MHI, hotspots in chlorophyll-*a* were observed along the northwest shorelines of Kaua‘i, O‘ahu, and Maui and the south shore of Moloka‘i (Fig 7). The greatest maximum anomalies were observed in the vicinity of Hilo on Hawai‘i Island, near Haleiwa along the northwest shore of O‘ahu, and much of the southwestern and northwestern shores of Kaua‘i. The lowest chlorophyll-*a* was observed along south Maui, west Lāna‘i, and the southeast and southwest coasts of Hawai‘i Island.

### Driver correlations across islands

**Fig 8:**
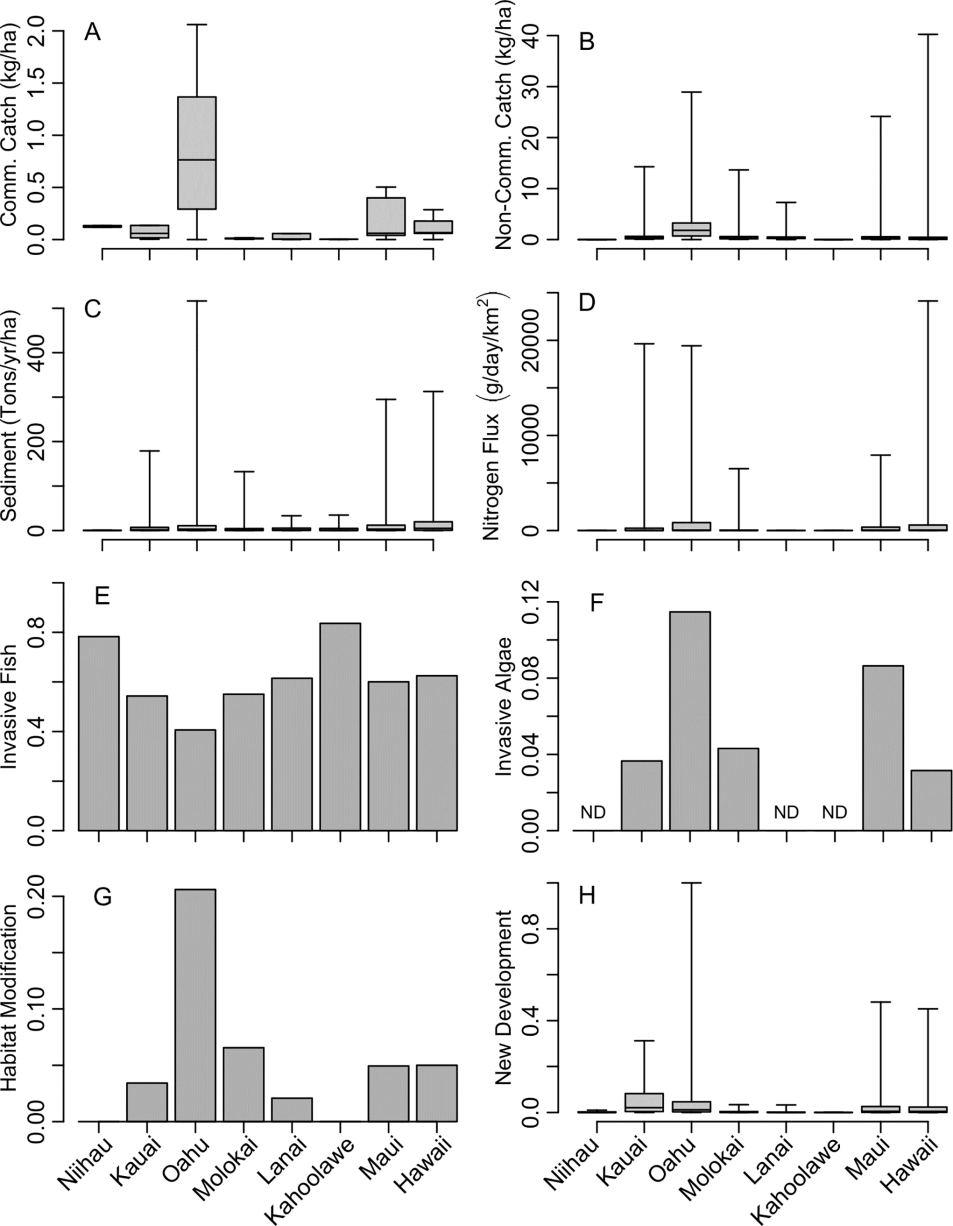
Primary anthropogenic drivers. Distributions of primary proximate anthropogenic drivers by island for the main Hawaiian Islands ordered from north to south. Box plots represent minimum, 1^st^ quartile, mean, 3^rd^ quartile, and maximum for each continuous driver, and categorical drivers (i.e. presence) are histograms of frequency of occurrence. Drivers include (A) total commercial catch for all gears combined (kg/ha), (B) total non-commercial catch for all gears combined (kg/ha), (C) sediment (Tons/yr/ha), (D) nitrogen flux from OSDS (g/day/km^2^), (E) invasive fish, (F) invasive algae, (G) habitat modification (proportion of reef area with presence), (H) new development (unitless).

Anthropogenic drivers were variable across islands, and were generally greatest around O‘ahu, the most densely populated island (Fig 8). Habitat modification on O‘ahu was correlated with the introduction of invasive algae and pressure from fishing. In addition, commercial fish catch was greatest and most variable around O‘ahu, and low around Kaho‘olawe, where catch for nearshore fish is highly restricted (Fig 8A). Across islands, non-commercial fish catch was more variable than commercial catch (Fig 8B), and overall ranges were as much as 20 times larger than commercial catch [50]. Sedimentation and nitrogen flux were also variable and had skewed distributions with extremely high values on O‘ahu, Maui, and Hawai‘i (Fig 8C and 8D). Invasive fish presence was frequent across all islands, and invasive algae were greatest on O‘ahu, followed by Maui (Fig 8E and 8F). Habitat modification was greatest on O‘ahu and was 5 times greater than any other island (Fig 8G). New development was also greatest on O‘ahu, however Kaua‘i had more widespread higher levels of development (median and 75^th^ quartile) compared to other islands (Fig 8H).

**Fig 9:**
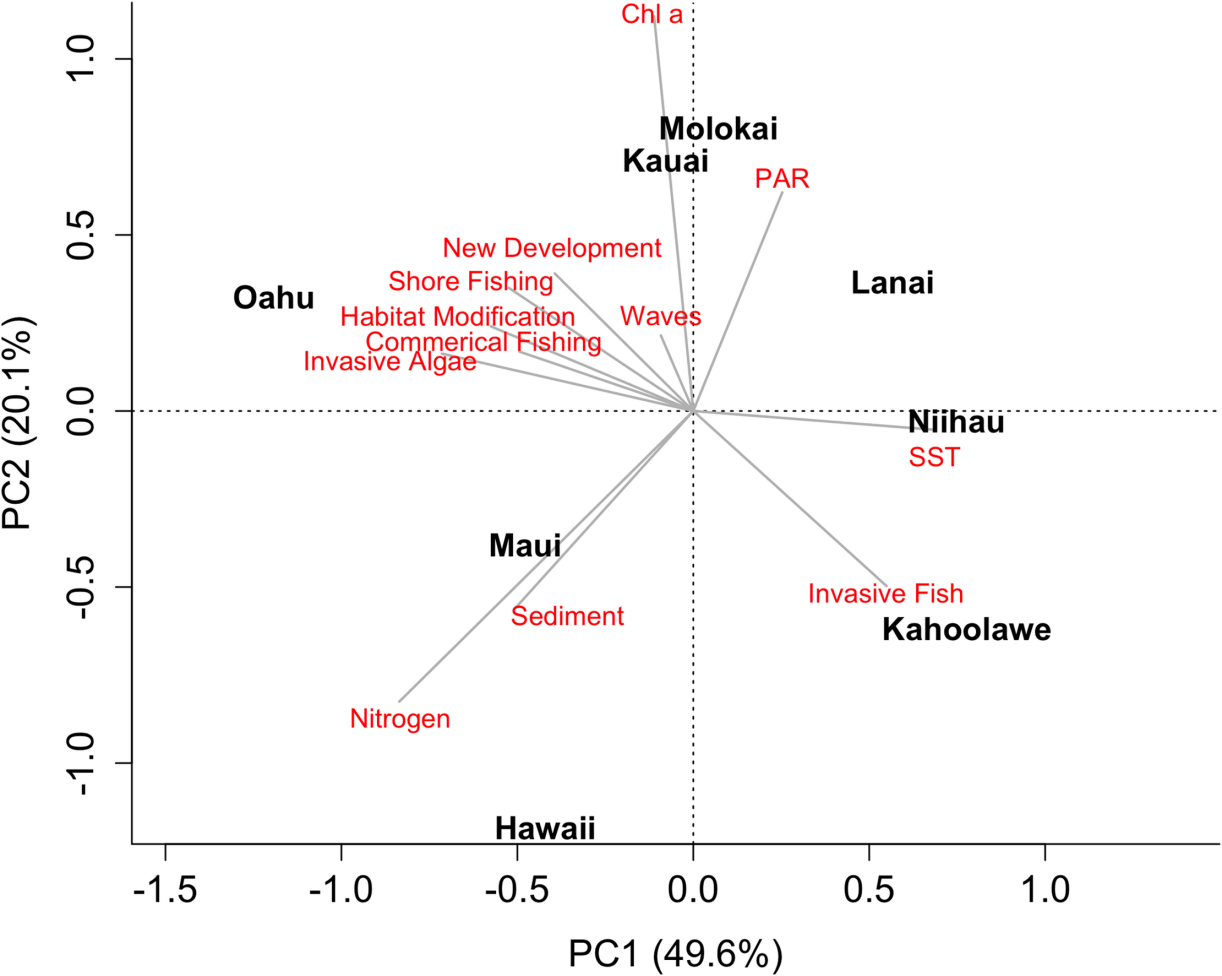
Principle component analysis of anthropogenic and environmental drivers. Principle component analysis (PCA) of the anthropogenic and environmental drivers based on median values by island. Loadings for each principle component drawn as grey lines in the direction of increasing values. PAR = Photosynthetically Active Radiation (irradiance); Chl a = Chlorophyll-*a*.

When both anthropogenic and environmental drivers were considered together in multidimensional space, islands clustered differently according to their correlation with particular variables (Fig 9). The first two axes of the PCA explained 69.7% of the variability contained in the 12 predictor variables used. The first axis (PC1), which was responsible for the majority of the variance explained (49.6%), clearly separated O‘ahu from all other islands, and was strongly correlated with fishing, habitat modification, new development, and invasive algae. Maui and Hawai‘i were also correlated with the same variables, but sediment and nutrients were also high and separated these two islands from the others. Notably, none of the environmental drivers were strongly associated with O‘ahu, whereas Kaua‘i, Moloka‘i, and Lāna‘i were all correlated with environmental drivers with higher values of chlorophyll-*a*, irradiance, and waves. Lāna‘i and Ni‘ihau were most correlated with SST. Kaho‘olawe was oriented opposite to O‘ahu, reflecting low values of all anthropogenic drivers except for high numbers of invasive fishes.

## Discussion

### Advancing spatial data integration

Integrating large and disparate data types presents new challenges in how we analyze, synthesize, and visualize information. However, the amount of data now available offers an unprecedented opportunity for understanding coral reefs and informing management decisions at multiple scales [82]. Our approach acknowledges that coral reef ecosystem dynamics are social-ecological, and driven by a combination of biotic processes, abiotic conditions and human drivers, and provides a novel integration of environmental and anthropogenic driver data across a regional coral reef system. This case study allowed us to establish and operationalize a methodological approach to spatially integrate data sets on coral reefs. The stepwise methodological approach we developed and conducted can be applied to future studies in order to leverage the power of extensive spatio-temporal data, inform pressing problems facing coral reefs, and better manage for ecosystem resilience to avoid coral reef tipping points.

The spatially explicit datasets on anthropogenic impacts in this study have advanced well beyond using just human population density and revealed a more explicit understanding of the spatial heterogeneity of human and natural drivers across a coral reef seascape. In addition, this work builds on recent coral reef anthropogenic driver advances by Cinner et al. [83] that incorporated distance to market and other social metrics, and provides a methodological approach to integrate environmental drivers. At a regional-scale, it is possible to assess proximate drivers with much greater accuracy, which is precluded in global analyses that often rely on proxy data. The regional-scale approach supports the understanding of complex dynamics governing ecosystem state, and therefore the policy interventions most likely to be successful using a place-based approach. With significant climatological changes predicted to occur in the coming decades, it is increasingly important to understand the major environmental and physical forces impacting coral reefs and the effects these environmental forces may have on the biology and management of the ecosystem. As a result, key natural environmental drivers, such as wave forcing, must be incorporated to more fully and accurately assess how these drivers relate to different reef states. Our synthesis and mapping of these comprehensive high-resolution spatial datasets of known key drivers of coral reef ecosystem state provides a pathway in understanding integrated social-ecological disturbance regimes.

### Anthropogenic drivers

#### Fisheries catch

The development of the fisheries catch layers provides an in-depth illustration of the design phase process of the methodological approach. The iterative engagement with experts and managers was a critical step to vet the fisheries catch spatial model and output. It is important to advance the mapping of fishing effort beyond simple proxies of human population density because severe declines in reef fish populations have long been documented for reefs both near and far from human population centers in Hawai‘i [31,20]. For example, in a recent global analysis of how coral reef fish biomass relates to the density of local human populations, high variability in fisheries conditions at low human population densities resulted in relatively weak explanatory models [24]. Our analytical approach in this study has progressed beyond a single proxy; by taking shoreline accessibility and boat access into account, we provide further levels of detail to visualize and map fishing effort by gear type across the State. Though human population was not used, island-scale demographics still play a role in the fisheries catch layers as O‘ahu has the highest boat ownership and the greatest number of launch ramps; thus, at the island scale, human population density does appear to be driving boat-based fisheries catch levels

The nearshore fisheries catch driver datasets provided an interesting spatial characterization of fishing across the MHI that highlighted unique contrasts in shore-based catch per unit area by distinct gear types. Accessibility is an especially important component when considering shoreline fishing in Hawai‘i, as limited availability of roads and steep cliffs drastically constrain fishing effort from shore in many areas across the state [20]. This is typically the case on Hawai‘i Island, and partly what drives the shore-based line and spear catch per unit area values higher than any other island across the state. Stamoulis et al. [84] showed the distribution of targeted reef fish biomass to be focused in remote and inaccessible areas across the Main Hawaiian Islands. Wave exposure on many NW facing shores can also have a significant effect on fishing accessibility, in particular large winter swells make north exposed shores inaccessible to fishing and make many of these areas de facto reserves for part of the year during high wave seasons [66,85–86].

These nearshore fisheries catch datasets reveal that very few locations in the Main Hawaiian Islands are exempt from fishing. No-take marine managed areas only account for 0.4% of nearshore (0–18m) waters in the state [87]. Large areas with little to no fishing pressure are those with restricted access such as the islands of Ni‘ihau and Kaho‘olawe, followed by inaccessible areas such as the North shore of Moloka‘i and portions of the NE (Hamakua) and SE coasts of Hawai‘i island. Not surprisingly, these same locations have been shown to harbor high biomass of targeted reef fishes [84].

The spatial patterns of shore-based fishing by gear type also highlight unique place-based preferences depending on target species and the dominant coastal habitats present that influence gear selection. Understanding and mapping the main gear types driving fish catch per unit area can support spatial management of fisheries across the islands by highlighting potential hotspots for management by gear type, bag or slot limits. McCoy [50] demonstrated that each fishing gear type in Hawai‘i targets distinct sets of species. The next steps for marine spatial planning applications could include a combination of this species-level knowledge, together with stakeholder input and the map outputs from this study in order to inform the designation of marine managed areas and implementation of harvest control rules for the conservation of key resource species (e.g., as proposed by Rassweiler et al. [88]).

#### Land-based stressors

Sediment is associated with nutrients and other forms of contaminants that are bound by organic matter and the iron-aluminum oxides that are typical of many of the highly weathered soils of the MHI. For instance, Wiegner et al. [89] found that 73% of the total phosphorus load and 43% of the total nitrogen load along windward Hawai‘i Island streams were bound to sediments. The coastal patterns of land-based pollution offer insight into where integrated land-sea management might be critical to achieving nearshore ecological goals (e.g., as evaluated in Maui by Oleson et al. [90]). In areas with acute land-based stressors, or in areas where land-based pollution might have direct, high economic costs (e.g., tourism areas), it may be necessary to mitigate the land-based stressors.

Generally, the windward (east-facing) streams with high sediment discharge on open coastlines have lower sediment residence times due to environmental conditions (e.g., wave energy, currents, bathymetry) compared to enclosed embayments. Residence time is important in the impact of sediment on reef quality in Hawai‘i [91]. For instance, we found Pelekane Bay on the western coast of Hawai‘i Island to have a high sediment load, which was derived from the adjacent watershed that discharges into a small, shallow harbor. This finding is supported by other recent field-based work in this area [92]. Research from elsewhere in Hawai‘i indicates that localized sediment plumes are common even during small storm events [93].

#### Environmental drivers

The Hawaiian Islands are exposed to large fluctuations in environmental forcings compared to other coral reef ecosystems across the Pacific Ocean [45]. Analysis of key environmental drivers presented herein indicates that even within the eight MHI, substantial gradients in drivers exist both among and within islands (Fig 7). For example, Hawai‘i receives wintertime ocean swells generated in the North Pacific that produce extremely large wave events (wave heights in excess of 7 m) several times in an average year [94]. Because these events are generated in the northwest, the northwesterly exposed coastlines receive the highest levels of wave forcing with many of the more sheltered coastal regions receiving much lower levels (an order of magnitude or more) of wave forcing. Variations in wave forcing influences important ecological processes such as coral reef development [95], and spatiotemporal patterning in benthic and reef fish communities [96]. Waves can also drive strong currents and nearshore mixing, which can influence sediment transport and resuspension [97], and reduce ocean temperatures during warming events [98].

Chlorophyll-*a*, a proxy for phytoplankton biomass and an indicator of phytoplankton production, exhibited large spatial variability across the MHI. Kaua‘i, O‘ahu and the region between Maui, Moloka‘i, and Lāna‘i showed long-term enhancements in chlorophyll-*a*, while a high frequency of anomalies were observed along south Moloka‘i, south Maui, and the northwest coast of Hawai‘i Island. These observed differences in chlorophyll-*a* were presumably indicative of changes in local nutrient concentrations, which can increase through a variety of natural process such as upwelling and mixing, and through human-related process such as agricultural run-off and poor waste-water management. Increases in phytoplankton biomass can impact multiple trophic groups within coral reef food-webs, promoting the development of calcium carbonate forming benthic organisms, namely scleractinian (hard) corals and crustose coralline algae [4], as well as the biomass of planktivorous and piscivorous fishes [4]. However, human-induced changes in phytoplankton biomass may result in negative ecological consequences, such as toxic algal blooms and coastal eutrophication [99].

### Linking science to policy and management through effective communication

These datasets will allow improved understanding of what drives variation in Hawaiian reefs and support management designed to promote reef resilience and protect reef ecosystem services. The spatial data synthesis from this project has been made publicly available to allow managers, researchers and members of the public to explore the data. In addition, this approach also serves to connect expert spatial analysts with new datasets, which will allow for future analysis and understanding of what drives variation on coral reefs. The goals of serving these data widely, and at no end-user cost, is to facilitate use of the data synthesis framework in further research and provide a scientific basis for improved policy and management actions at the state level through engagement with Hawai‘i Division of Aquatic Resources (DAR) and other management agencies. Our project is sharing information and building connections in Hawai‘i through a variety of flexible formats, including story mapping and data visualizations. The science communication approach was implemented in order to bridge the technology barrier by allowing users to easily explore mapped data without previous mapping technology experience.

We have also distilled our scientific outputs and served this information across several platforms that allow for visualization of mapped reef drivers in an interactive, user-friendly online mapping interface as well as static map products (www.pacioos.hawaii.edu/projects/oceantippingpoints). Our approach will allow scientists, policy-makers, and community members to access information in the format that meets their analytical or information needs. For example, the NOAA Integrated Ecosystem Assessment program is currently leveraging these datasets to investigate temporal trends in coral reef monitoring data and guide research in identifying local drivers that undermine or promote coral reef health and resilience on the west coast of Hawai‘i Island, which in-turn supports current management decision efforts by local State agencies. Specifically, NOAA is investigating what drivers best explain temporal trends in monitoring data, as well as patterns of bleaching and recovery from the 2014/2015 mass bleaching events.

### Applications to support spatial management and policy

Currently, less than 0.4% of coral reef ecosystems in the main Hawaiian Islands are protected through no-take marine protected areas (MPAs) [100, 87]. Advancing coastal management in Hawai‘i will involve developing tailored management strategies to control key stressors such that nearshore ecosystems and the ecosystem goods and services they supply are sustained. To move toward more effective marine resource management, there is a need to integrate ‘place-based’ management approaches together with holistic marine spatial planning that ensures patterns of connectivity and disturbance are managed across the seascapes [101–103]. One of the first steps in such marine spatial planning efforts involves mapping and integrating biophysical and human dimensions, including drivers and human uses, across the entire ecosystem [104–107]. However, the lack of comprehensive, spatially explicit data and spatial data integration methods can impede holistic, ecosystem-scale ocean planning and area-based management [82]. Our research reveals a pragmatic approach to assess these complex drivers and their spatial distribution and intensity across a regional seascape. While our focus has been on the nearshore reef environment, additional anthropogenic stressors, like pelagic fisheries, commercial shipping, recreational activity, marine debris, and military activity also need to be mapped to support marine spatial planning in intertidal and deeper habitats. Further, analysis could also examine how these anthropogenic and environmental drivers interact, as well as integrating anthropogenic impact drivers together with information on social benefits from seascapes (ecosystem services) [108, 104].

While strength of this methodological approach will vary based on the available data sets, equally critical is the success of stakeholder engagement and the distillation and synthesis involved in science communication and outreach efforts. Data collected for research purposes often fails to meet decision-maker needs, e.g., by ignoring management constraints or stakeholder objectives, and thus is rarely used to affect decisions [109]. The true utility of harnessing the power of large data sets lies in the distillation of these data into knowledge in a way that can effectively provide the best available science to inform management and policy. Ideally, each step in the methodological process will involve iterative discussion and engagement with stakeholders and end users (e.g., managers, policy-makers) to address their individual goals and needs, enabling development of a final product that is ready for implementation by management and uptake by the stakeholder community [88]. Co-creation of knowledge by researchers, data users and stakeholders is fundamental to ensuring that knowledge is provided in metrics that resonate with stakeholder objectives, is in a format that can be analyzed by end users for guiding decisions (e.g., via tradeoff analysis; [110–111]), and will remain relevant for adaptive governance [109,112–113]. Managers and decision-makers in Hawai‘i recognize the need for data generally and ecosystem services knowledge specifically [114–115], and have been intimately involved in every step. Iterative engagement with the end users and stakeholders is built into the methods outlined here, allowing their needs and objectives to be identified, reviewed, and ultimately addressed. Accurate, publicly available data provide transparency and support effective, timely, science-based decision-making, which should lead to more effective management of these valuable resources that mean so much to so many.

## Supporting information

S1 Supplement Driver data analysis, assumptions, and limitations. (PDF)

S2 Supplement Detailed GIS methods used to create fisheries catch maps. (PDF)

S3 Supplement Correlation matrix for driver layers used in PCA. (XLSX)
