# Correction: Advancing the integration of spatial data to map human and natural drivers on coral reefs

**DOI:** 10.1371/journal.pone.0204760

**Published:** 2018-09-24

**Authors:** The PLOS ONE Staff

## Notice of republication

Incorrect versions of Supporting Information files S1 Supplement and S2 Supplement were published in error. The publisher apologizes for the error. This article was republished on September 14, 2018 to correct for the error. Please download this article again to view the correct version.
