# Supplementary material for: Advancing the integration of spatial data to map human and natural drivers on coral reefs
Source: PLoS One. 2018 Mar 1;13(3):e0189792. doi: 10.1371/journal.pone.0189792 (PMC5832214; doi:10.1371/journal.pone.0189792)
Supplement: S1 Supplement — (PDF) [file pone.0189792.s001.pdf]

SUPPLEMENT S1

Data analysis, assumptions and limitations

Fisheries Catch

The accessibility model in this study assumes all nearshore habitat is equal and that fisherman do not preferentially target areas with higher quality habitat or higher fish biomass. The relative weighting across different shoreline accessibility classes is guided by expert input, but no quantitative information exists to directly inform the models. Potential improvements to the fisheries catch model could incorporate travel time to boat harbors and ramps (as in [1]) in addition to surrounding population density, as well as statistics on the average number of boats using each facility per year (info for the latter currently does not exist). Wave exposure is an important seasonal component to shoreline and boat accessibility and this was not accounted for in our effort to map shore-based and boat-based fish catch. However, information on seasonal catch was unavailable and it is unknown whether average annual catch on northern exposed shores would be lower because of this temporal exclusion. In addition, if fishing increases on north facing shores during the summer months when the waters are calm, there may be no net effect on annual fisheries catch. In this study, it was assumed that greater fisheries catch was characteristic of more accessible fishing areas, but there are cases that may not satisfy this assumption. For example, a remote fishing grounds where additional effort is placed towards access because the CPUE is high could possibly have higher than predicted annual catch. Kīholo Bay on Hawaiʻi Island is one example where our maps underestimated catch compared to a creel survey. Alternatively, highly accessible but overfished locations are present throughout the state where our maps overestimate annual catch (e.g., south shore Oahu). We accounted for spatial fishery management to the greatest extent possible, but limitations stem from discrepancies

between gear- and species- specific regulations and the gear groupings for which catch estimates were available (line, net, and spear). Areas where an entire gear group is completely prohibited were set to zero catch (assuming 100% compliance with regulations). However, areas with regulations on specific gears within a gear group or certain species could not be accounted for. For example, there are areas where lay nets are banned but thrownets and other types of nets area allowed, and there are marine managed areas where line fishing is allowed but the type and number of lines is restricted. Similarly, there are managed areas that do not restrict any gears but limit or prohibit take of certain species. To more accurately reflect all the nuances of fishery management in maps of catch, estimates by more specific gears and by species would be needed. While McCoy [2] presents species level estimates we did not have access to these at the time of model development, and there are relatively few areas in the MHI with species-specific prohibitions.

Despite these limitations, we were able to successfully validate the maps of non-commercial catch with the limited amount of creel survey information that exists (Table A; Figure A). The intricate methodology developed to map non-commercial fishing was vetted with DAR and NOAA resource experts on multiple islands throughout the process. In addition, the final non-commercial reef fish fisheries maps were validated with estimates of non-commercial catch at seven sites across the state where creel surveys have been conducted. The successful validation of the fishing layer developed for this project underscores the usefulness of this dataset as a stand-alone product for future research applications like exploring intra-island patterns in reef fish biomass to better direct fisheries management and enforcement resources.

Land-based Stressors

We used the outputs from Falinski's [3] modified InVEST Sediment Delivery Ratio (SDR) estimates of sediment export across the Main Hawaiian Islands. The InVEST SDR has shown to be sensitive to the scale of the input data, but with calibration has successfully predicted sediment export across a range of climatic conditions[4]. Although the model was calibrated to total export, the processes modeled only capture hillslope erosion, and do not represent contributions from mass wasting, gully erosion and streambank erosion. In particular, areas that were once dominated by monocultures of sugarcane or pineapple have contributed to build-up of fine sediment along the banks of gulches and channels that takes decades and centuries to export via bank erosion [5]. Additionally, in highly degraded areas like Kahoʻolawe or northeastern Lānaʻi, decades of overgrazing have left gullied surfaces eroded to the bedrock that would not be well represented by the model. Lastly, the model input for forest type is "Evergreen Forest", which does not distinguish between healthy native forests (for instance northeast Maui), or forests dominated by invasive species (such as windward Oʻahu). Further work that correlates forest disturbance to erosion rates would assist in refining the model estimates of sediment.

Our study spatially modeled the dispersion of sediment loads offshore by using a relatively simple kernel function. Wave action, currents, and resuspension are all important factors in sediment impacts on reefs. One limitation of the ocean circulation models available in coastal Hawaiian waters was the lack of models at the appropriate spatial and temporal scales to incorporate dispersal by dominant current direction. As new data becomes available this will be an important future step for improving the mapping of sediment dispersal offshore.

The key limitation for the nutrient layer is that it only considers on-site waste disposal and in many watersheds, agriculture, pastures, golf courses and injection wells also contribute to the

nutrient loads. In fact, preliminary analyses suggest that cesspools are only 25% of the total nutrient budget in groundwater. The estimated values of nitrogen flux and phosphorous flux were based on Tax Map Key parcels with onsite waste disposal systems. Nutrients from municipal treatment plants and injection wells were not captured here due to data limitations, nor were nutrients from surface runoff and infiltration (e.g., from fertilizer and animal waste). Expanded data on all land-based pollution sources would improve estimates of the total loads reaching coral reefs. Similar to the sediment load layer improvement, better modeling of nearshore circulation and biogeochemical processes could greatly improve the final product.

Invasive species

The map outputs of invasive fish and algae are presence-only, as the status in un-surveyed areas is unknown and there is the potential that a survey failed to observe an invasive species where it is actually present. While large gaps exist, they do not necessarily indicate that these species are absent from those areas but instead could indicate that no data exists, or species were not recorded on existing transect data. For example, the northern tip of Oahu (Kahuku / Turtle bay) is a gap in monitoring data but the North east side (Kahana to Lāʻie) has fairly good data coverage with no recorded sightings of invasive species. Future work could try to map abundance of invasive species but would need to clearly distinguish which areas there are no data vs areas with confirmed absence of invasives.

Habitat degradation

A caveat in the habitat degradation spatial layer is that structures that have been around for 100+ years (e.g., fishpond walls) and have cultural value, are not differentiated in the source data from new structures (e.g., a seawall or pier) constructed in the last decade. In addition, there may be different ecological impacts from different types of habitat modification – a seawall vs dredging

– but this study combined them based on our definition of habitat modification as the alteration or removal of geomorphic structure as a result of human use.

Limitations and caveats with environmental driver data

Sea Surface Temperature (SST)

Three SST datasets are concatenated to provide continuous, gap-free ocean temperature data from 1985 - 2013. The dataset concatenation applied a bias adjustment, derived from linear regression to the overlapping periods of each of the data sets. The following represent the analysis steps:

Step 1: Production of weekly composite, gap-filled SST data from the NOAA Pathfinder v5.2 SST 1/24° (~4 km), daily dataset for each location. This dataset covers the period January 1985 – December 2012 at the native spatial resolution (i.e., ~4 km).

Step 2: Production of weekly composite SST data from NOAA's Center for Satellite Applications and Research blended SST 0.1° (~11 km), daily dataset. This dataset covers the period February 2009 – October 2013 at the native spatial resolution (i.e., ~11 km).

Step 3: Production of weekly composite SST data from NOAA's Center for Satellite Applications and Research blended SST 0.05° (~5 km), daily dataset. This dataset covers the period March 2012 – December 2014 at the native spatial resolution (i.e., ~5 km).

Step 4: Using the overlap period between datasets, we linearly regress paired (in time) data to determine the bias between datasets for each location. We then bias-adjust the datasets to represent the 5 km dataset and blend the datasets through overlap periods to complete a single SST time series dataset covering 1985 – 2013 for each location.

Chlorophyll-a and Irradiance

Satellite-derived ocean color algorithms are calibrated for optically-deep waters, where the signal received by the satellite sensor originates from the water column without any bottom contribution. In our study region, optically-deep waters are typically deeper than 30 m[6]. In optically-shallow waters such as lagoons, regions within atolls, and most coral reef environments, bottom substrate properties and sediment suspension may affect light propagation, which increases marine reflectance and data quality issues when quantifying in-water constituents, such as chlorophyll-*a* [7].

Satellite-derived irradiance, specifically photosynthetically available radiation (PAR; defined as downwelling irradiance between 400 and 700 nm), is subject to similar data quality concerns. The data production algorithm[8], in addition to a number of other quality control steps, incorporates irradiance attenuation in the overall calculation of irradiance. Attenuation sources in the atmosphere include the absorption and scattering of irradiance as a result of concentrations of ozone, water vapor, and aerosols. Attenuation sources at the air-sea interface include reflection, associated with surface properties such as sea-surface roughness and levels of sea foam [8]. Optically-shallow areas are often wrongly interpreted as irradiance attenuation sources, thereby leading to spuriously low irradiance values [8].

Taking into account the data-quality concerns described above, we developed a multistep masking routine to remove contaminated data pixels (sensu [9]). We used the 30-m contour as the cutoff for satellite pixel inclusion; all pixels inshore of the 30-m isobath were identified and removed from the data set prior to analysis. This step, however, is not sufficient to ensure error-free chlorophyll-*a* and irradiance data sets, because pixels outside the 30-m isobath may still contain biased information associated with optically-shallow waters. This occurs because data

pixels are box-like in shape and are georeferenced at their center point; thus, information contributing to any single pixel value is collected up to one-half a pixel diagonal distance away. To address this, we created a data exclusion zone of one-half a pixel diagonal in length (0.0295° or ~3.27 km) everywhere perpendicular to the 30-m isobath, with all pixels on or within this zone also removed from the data set.

Wave Power

Small-scale nearshore processes and rapid changes in wave refraction, amplification and dissipation were poorly resolved in the University of Hawai‘i’s wave model, resulting in anomalously high wave forcing values along the coastline. As such, we removed the nearest wave model pixels to shore, or all pixels 500 m or closer to shore across all islands. Therefore, actual wave power values presented herein are likely a conservative estimate of the actual wave forcing experienced across the Hawaiian Islands. For wave model assumptions and limitations, please see Li et al. [10].

Table A. Creel survey data sources:

Location	Island	Survey Period	duration	Type	Citation
Hanalei*	Kaua‘i	6/1992-12/1993	1 year, 4 mo.	Final Report	[11]
Waikiki*	O‘ahu	6/1998-8/2001	1 year, 2 mo.	Dissertation	[12]
Kahekili	Maui	1/2011-12/2011	1 year	Final Report	[13]
Wailuku	Maui	3/2013 - 5/2014	1 year	Final Report	[14]
Maunalei**	Lāna‘i	5/2013 - 6/2013	2 weeks	Frame Survey	[15]
Kaupulehu	Hawai‘i	8/2013-8/2014	1 year	Final Report	[16]
Kiholo	Hawai‘i	5/2012-5/2013	1 year	Publication	[17]

Figure A. Fisheries catch mapping validation. Scatter plot of annual catch estimates from creel surveys on the y-axis vs. annual catch of reef fish for corresponding locations from the noncommercial shore-based total catch map layer on the x-axis. The grey dashed line has a slope of one - a point falling on this line would indicate a perfect match. The solid black line is a fitted linear regression with intercept anchored at (0,0) ($p < 0.01$). R^2 and slope of line are reported on the graph.

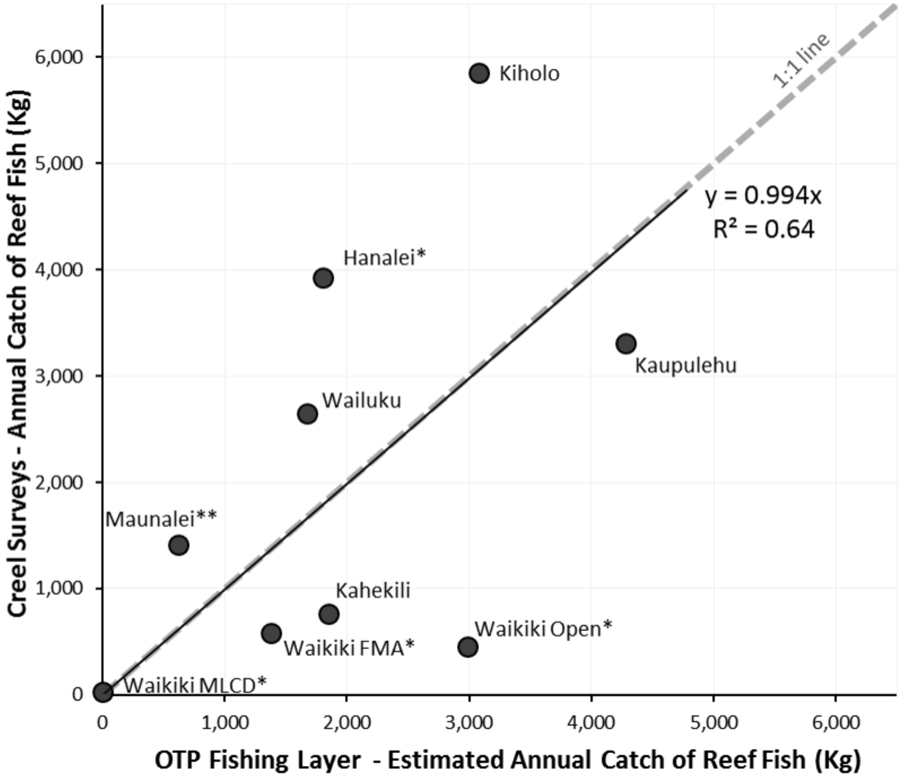

* creel surveys conducted outside the time frame of Marine Recreational Information Program (MRIP) data used to derive statewide fishing layer (2004 – 2013)

** data for Maunalei is from a two week frame survey, not a full creel survey

References:

1. Cinner JE, Huchery C, MacNeil MA, Graham NAJ, McClanahan TR, Maina J, et al. Bright spots among the world's coral reefs. Nature. 2016;535. doi:10.1038/nature18607
2. McCoy KS. Estimating Nearshore Fisheries Catch for the Main Hawaiian Islands. University of Hawaii at Manoa - M.S. Thesis. 2015.
3. Falinski KA. Predicting sediment export into tropical coastal ecosystems to support ridge to reef management. University of Hawaii at Manoa - Ph.D. Dissertation. 2016.
4. Hamel P, Falinski K, Sharp R, Auerbach DA, Sánchez-Canales M, Dennedy-Frank PJ. Sediment delivery modeling in practice: Comparing the effects of watershed characteristics and data resolution across hydroclimatic regions. Sci Total Environ. 2017;580: 1381–1388. doi:10.1016/j.scitotenv.2016.12.103
5. Stock JD, Falinski KA, Callender T. Reconnaissance sediment budget for selected watersheds of West Maui, Hawai ʻi. US Geological Survey; 2016.
6. Mumby PJ, Skirving W, Strong AE, Hardy JT, LeDrew EF, Hochberg EJ, et al. Remote sensing of coral reefs and their physical environment. Mar Pollut Bull. 2004;48: 219–228. doi:10.1016/j.marpolbul.2003.10.031
7. Boss E, Zaneveld JR V. The effect of bottom substrate on inherent optical properties: Evidence of biogeochemical processes. Limnol Oceanogr. 2003;48: 346–354. doi:10.4319/lo.2003.48.1_part_2.0346
8. Carder KL, Chen FR, Hawes SK. Instantaneous photosynthetically available radiation and absorbed radiation by phytoplankton. NASA Ocean Color web page. 2003; 1–24. Available: https://modis.gsfc.nasa.gov/data/atbd/atbd_mod20.pdf
9. Gove JM, Williams GJ, McManus MA, Heron SF, Sandin SA, Vetter OJ, et al. Quantifying Climatological Ranges and Anomalies for Pacific Coral Reef Ecosystems. PLoS One. 2013;8. doi:10.1371/journal.pone.0061974
10. Li N, Cheung KF, Stopa JE, Hsiao F, Chen YL, Vega L, et al. Thirty-four years of Hawaii wave hindcast from downscaling of climate forecast system reanalysis. Ocean Model. 2016;100: 78–95. doi:10.1016/j.ocemod.2016.02.001
11. Friedlander AM, Parrish JD, Peterson JD. A survey of the Fisheries of Hanalei Bay, Kauai. Report to Hawaii Division of Aquatic Resources. Honolulu, HI; 1995.
12. Meyer CG. An Empirical Evaluation of the Design and Function of a small marine reserve (Waikiki Marine Life Conservation District). University of Hawaii, Ph.D. Dissertation. 2003.
13. Friedlander AM, Koike H, Kekoa L, Sparks R. Design, development, and implementation of a survey of the fisheries of the Kahekili Herbivore Fisheries Management Area. Final Report to Hawaii Division of Aquatic Resources. 2012.
14. Koike H, Carpio J, Friedlander AM. Final Creel Survey Report for Wailuku Community Management Area , Maui County , Hawaii. Final Report to Conservation International. 2014.

15. Koike H, Kaho’ohalahala S, Friedlander AM. Frame Creel Survey Report for Maunalei, Lanai. 2013.

16. Koike H, Wiggins C, Most R, Conklin E, Minton D, Friedlander A. Final Creel Survey Report for Kaʻūpūlehu Creel Survey Project, North Kona, Hawaiʻi Island. Final Report to The Nature Conservancy. 2015.

17. Kittinger JN, Teneva LT, Koike H, Stamoulis KA, Kittinger DS, Oleson KLL, et al. From reef to table: Social and ecological factors affecting coral reef fisheries, artisanal seafood supply chains, and seafood security. PLoS One. 2015;10: 1–24. doi:10.1371/journal.pone.0123856
