# Supplementary material for: Advancing the integration of spatial data to map human and natural drivers on coral reefs
Source: PLoS One. 2018 Mar 1;13(3):e0189792. doi: 10.1371/journal.pone.0189792 (PMC5832214; doi:10.1371/journal.pone.0189792)

SUPPLEMENT S2 - Detailed GIS Methods for Fisheries Catch Mapping

The methodology developed for each fishing data layer is detailed below including the data sources and processing steps. All analysis was performed in ArcGIS 10.1 or R 3.1.x. All spatial data were projected to UTM zone 4 North, NAD 1983. All data layers use a common shoreline derived from NOAA Habitat Maps (from 2007 IKONOS imagery) and NOAA Continually Updated Shoreline Product (CUSP), with certain sections of coastline (e.g. Wai'opae Tide Pools) hand edited based on ESRI world imagery basemap and WorldView 2 imagery.

We used 2 primary sources of catch data as the basis for our fishing maps: Commercial marine landings (CML) data from the State of Hawaii Division of Aquatic Resources (DAR), and estimates of non-commercial catch from McCoy et al. (2017, in prep) derived from Marine Recreational Information Program (MRIP) data.

Commercial Fishing

Commercial catch data received from DAR spanned an 11-year period from 2003 through 2013 and is aggregated by year, reporting block, gear, and species. DAR filters CML data before release such that reporting blocks with less than three fishers reporting are excluded, in order to protect fisher identities. It is not possible to explicitly distinguish between boat-based and shore-based fishing with the gear types reported in CML data. We filtered the data for reef fish species only (Table A), and calculated average annual catch in kilograms by reporting block and gear groupings that matched the MRIP data (line, net, and spear) (Table B). Spatial footprints of inshore commercial reporting blocks were obtained from the shapefile served on the Hawaii Statewide GIS Program website (http://planning.hawaii.gov/gis/ ; filename: Fishchart2008.shp).

Annual catch values were joined to reporting blocks using the Area ID. Using the Polygon to Raster conversion tool, average annual commercial catch data was converted from polygon to raster with 100 m resolution for each gear type. Map pixels within marine protected areas that are full no-take, explicitly prohibit commercial fishing, or prohibit specific gear groupings, were set to zero respectively for each gear layer. Catch in Defacto MPAs and other areas with restricted access were reduced according to expert input and local knowledge. Next each map pixel was divided by the number of raster cells within each reporting block so that units are comparable to Non-Commercial fishing layers (kg/ha). The result assumes commercial catch is evenly distributed spatially across each reporting block.

Non-Commercial Fishing

McCoy et al. (2017, in prep) estimated average annual non-commercial catch of reef fish for the years 2004 – 2013 for the 6 most populated Main Hawaiian Islands (Kauai, Oahu, Molokai, Lanai, Maui, and Hawaii), by platform (boat, shore) and gear (line, net, spear). McCoy's estimates also represent catch of only reef fin fish. Many more reef fish species were recorded in MRIP data compared to CML – see McCoy (2015) for species lists and specific gears.

Shore-based fishing

To quantify boat-based fishing at a within-island spatial resolution we combined MRIP estimates with two measures of shoreline accessibility (steepness and presence of roads), and gear specific footprints.

Slope of the shoreline was calculated in degrees using the USGS 10 m Digital Elevation Model (DEM). Then focal statistics was used to calculate the average slope within a 100 m radius of each pixel. Next the string of raster cells that fall along the coastline were extracted and reclassified into 3 categories (Table C). Exploration of the data showed that 0-3 degrees average slope characterize coastline that would be easily accessible to anyone, 3-20 degrees includes areas that are more difficult but possible to access and fish from, and average slope greater than 20 degrees is very rugged coastline that is not possible to access and high cliffs that are too tall to fish off of (Fig A1).

Next, the coastal raster cells with associated slope information were converted from Raster to Point data and a Near Analysis was used to calculate the distance within 1 km to the nearest roads of various type. Topologically Integrated Geographic Encoding and Referencing (TIGER) road data from the US Census Bureau were used based on completeness compared to other road data available from the Hawaii Statewide GIS Program. TIGER roads are classified into 14 types of roads, which we further grouped into 3 classes: 1) paved public roads, 2) 4WD roads, and 3) private roads and foot trails (Private roads and foot trails were grouped into a single class because both are relatively rare across the dataset and both are believed to represent a much lower level of accessibility than public and 4WD roads). Coastal points were classified by presence and type of roads within distances of 100 m, 200 m, 500 m, and 1 km. Type of roads present were determined using a priority ranking based on ease of accessibility with paved public roads ranking highest, 4WD roads next, and private roads and foot trails ranked as lowest (Table D). For example, if all road types are present within 500 m of a point, that point would be assigned “paved public road” because that ranks highest in ease of accessibility, regardless of which road type was closest to the shore. Final map layers were created using the 500 m cutoff,

as this layer provided the most precise information without compromising our ability to make conclusions about this proxy for coastal access (Fig A2).

Attributes for slope and road accessibility were then combined into a single accessibility criteria. A weighting scheme was created that assumes easily accessible shorelines with flat slopes and paved public road access have the highest catch, and therefore the highest weight, and that catch and weight decreases incrementally with level of accessibility (Table E). Any combination that includes no accessibility due to steep slopes received a zero weight (and therefore zero fishing). Weights sum to 1. These weights were then multiplied by the MRIP island-scale estimates of annual catch at each coastal point, for each of the three shore-based gear types: line, net, spear.

For Line fishing, catch was extended offshore a distance of 200 m. For Net fishing, catch was extended offshore to the 20 ft (6.1 m) depth contour, with a maximum distance from shore of 1 km. For Spearfishing, a logistic decay function was used so catch decreases with depth to 40 m or a maximum distance of 2 km from shore (Fig B). The equation for spearfishing decay with depth is as follows:

$$C_p = \frac{C_i}{1 + e^{(-0.26\cdot(D+20))}}$$

Where C_p is relative catch at a given pixel, C_i is the island-scale catch estimate weighted by shoreline accessibility, and D is depth in meters.

Next, Marine Managed Areas and de facto MPAs were accounted for by conducting a comprehensive review of regulations and boundaries. In full no-take MPAs catch by gear was set to zero and in other areas with restricted access catch was reduced according to expert input and local knowledge. Finally, pixel values were rescaled to be in units of kg/ha such that all cells

within an island's shore-fishing footprint sum to the value of the original MRIP island-scale estimate. Units, pixel size and grid alignment are consistent with all other fishing layers so that they can be compared directly or added together for various uses.

Boat based fishing

To quantify boat-based fishing at a within-island spatial resolution we combined MRIP estimates with distance from boat launches and a Gaussian decay function that assumed the majority of the catch occurs within 15 – 20 km of each harbor (Fig A3). Additionally, we weighted boat harbors by the human population present within 30 km, and accounted for marine managed areas and restricted access areas (de facto MPAs e.g. Military Danger Zones). First, point data for boat harbors and launch ramps were combined from two datasets available from the Hawaii Statewide GIS Program website (filenames: Harbors.shp and BoatingFacilities.shp). Data were checked for quality and updated as necessary to ensure only operational boat harbors and launch ramps were included, and geographic positions were accurate. Anchorages, fishing piers, historic, and disused ramps/harbors were removed prior to analysis.

Boat facility weighting factors were calculated based on total human population within 30 km of each boat harbor or ramp. Human population was mapped based on 2010 census data and LANDFIRE land use/land cover data using the USGS Dasymetric Mapping Tool to gain a more accurate representation of population distribution. A 30 km buffer was then created around each boating facility and the Zonal Statistics tool was used to sum the human population within each buffer. These population values were then used to assign weights to each boating facility in order

to allocate a proportion of total island catch estimates to each boat harbor or ramp (more described below). These weights sum to 1 for each island.

Next, boating facility cost allocation footprints were created by calculating distance to boating facility using the Cost Allocation tool iteratively for each boat harbor/ramp, with a maximum distance of 80 km. This allows for fishing influence from one harbor to overlap with nearby ones as well as with neighboring islands. A cost surface was created by converting island polygons to a 100 m raster with land pixels assigned a value of 1,000,000, and ocean pixels a value of 1. During rasterization, priority was set in the Polygon to Raster tool for ocean areas - this ensures that boating facility points do not fall on land. The cost distance surface output shows the distance from the nearest ramp/harbor to a given pixel without traveling over land. The resulting raster was then clipped to the footprint of inshore commercial reporting blocks.

In order to allocate catch proportionally to each boat harbor/ramp, estimated annual catch at the island scale and the human population based weighting factors were joined to the attribute table of each boating facility's cost allocation footprint, and used in a Gaussian decay function with each distance surface. The equation for the decay function is as follows:

$$C_p = C_i \cdot e^{\left(\frac{-1 \cdot d^2}{10^9}\right)}$$

Where C_p is the relative catch at a given map pixel, C_i is the island-scale catch estimate, and d is distance to boat harbor in meters (Fig A3).

This decay function assumes the majority of catch occurs within 15-20 km of a harbor or ramp and declines more rapidly beyond that. Catch in full no-take MPAs, and marine managed areas

that prohibit boats or entire gear groups were set to zero respectively for each gear specific layer. Other areas with restricted access were reduced according to expert input and local knowledge. Pixel values within each boating facility's footprint were then rescaled such that the sum in each footprint was equal to the respective boat facility's weighting factor times the MRIP catch estimate for that island in units of kg per pixel (kg/ha). Finally, all raster layers for each boat harbor/ramp were summed together using the Cell Statistics tool.

Final pixels values are in units of kg / ha such that the sum of all pixels for each island is equal to the original MRIP island-scale estimate. The spatial footprint for boat-based layers is the same as inshore reporting blocks for commercial catch. Units, pixel size and grid alignment are consistent with all other fishing layers so that they can be compared directly or added together for various uses.

Tables and Figures

Table A. All species reported in commercial catch data (CML) and the classification used to filter data to only reef finfish species.

Reef Species	Reef Spp. (cont'd)	Non-reef Species	Non-Reef Spp. (cont'd)
Aawa	Munu	Aku	Ogo
Ahaaha	Naenae	Akule	Onaga
Aholehole	Nenue	Alfonsin	Ono
Alaihe	Nohu	Aweoweo (deepsea)	Opakapaka
Alaihe mama	Nunu	Bigeye tuna	Opelu
Amaama	Oio	Billfish-misc.	Opihi alinalina
Api	Olililepa	Black coral	Opihi makaiauli
Awa	Omaka	Black marlin	Peles murex
Awaawa	Omilu	Blue marlin	Sailfish
Aweoweo	Opelu kala	Day tako	Samoan crab
Black kole	Pakuikui (tang)	Ehu	Shark-misc.
Butaguchi ulua	Palani	Gaskoins cowry	Short-nosed spearfish
Dobe ulua	Panuhunuhu	Gindai	Spiny green lobster
Ea (wrasse)	Panunu	Golden kali	Spiny red lobster
Gunkan ulua	Paopao ulua	Granulated cowry	Striped marlin
Hahalalu	Papa ulua	Hapuupuu	Swordfish
Hinalea	Poopaa	Hee (octopus)	Thresher shark
Humuhumu	Pualu	Hogo	Tiger shark
Kagami ulua	Puhi eel-misc.	Kahala	Tilapia
Kaku	Puhi white	Kalekale	Tombo
Kala	Roi	Kamanu	Tuna-misc.
Kawelea	Sasa ulua	Kawakawa	Walu
Kole	Taape	Keokeo	Yellowfin tuna
Kumu	Toau	Kona crab	Yellow-tail kali
Kupipi	Uhu parrot-misc.	Kuahonu crab	
Lae	Uku	Laevigatus shrimp	
Laenihi	Ulua-misc.	Lehi	
Maiko	Umaumalei	Limu kohu	
Malu	Uouoa	Limu wawaeiole	
Manini	Wahanui	Limu-misc.	
Maomao	Weke	Mahimahi	
Menpachi	Weke aa	Mako	
Moana	Weke nono	Miscellaneous	
Moana kale	Weke pueo	Monchong	
Moi	Weke ula	Muhee (squid)	
Mu	White ulua	Oceanic whitetip	

Table B. Gear types reported in CML data and their corresponding classification to match with MRIP gears. *Asterisks indicate gears for which there is zero catch after filtering the species to only reef fish.

CML Method	MRIP Equivalent
Aku boat*	Line
Casting	Line
DSHL	Line
ISHL	Line
Kaka line	Line
Shortline*	Line
Troll	Line
Tuna HL	Line
Vertical line*	Line
Net	Net
Dive/spear	Spear
Other*	Other
Trap	Other
Handpick*	Other

Additional notes:

- Kaka line - only one block reporting (300lbs of Menpachi)
- Tuna HL catch of reef fish was almost 100% Uku
- Line gears: Dominant species reported were Uku, Menpachi, and Taape
- Dive/Spear: dominant catch is Uhu
- Net: dominant catch is Manini, Aholehole, and Weke ula
- Trap fishing only reported on Oahu (dominant catch = Uhu and Palani)

Table C. Shoreline accessibility in term of shoreline steepness, calculated as the average slope within 100m inland of the shoreline.

Accessibility	Slope
High accessibility*	0 – 3 °
Low accessibility*	3 -20 °
No accessibility	> 20 °

*3° slope over 100m ≈ elevation gain of 5 m (17 ft)
*20° slope over 100m ≈ elevation gain of 36 m (120 ft)

Table D. Shoreline Accessibility by type of road within 500m of coastline.

Accessibility	Road Type
High accessibility	Paved Public Roads
Less accessibility	4WD roads
Least accessibility	Private road or foot
No accessibility	None

Table E. Weighting scheme for shore based fishing, combining shoreline steepness and type of road near the shoreline. Weights sum to 1.

		**Road within 500m of shore**			
		Paved Public	4WD Road	Private	none
**Slope Access**	High	0.4	0.15	0.05	0.02
	Low	0.25	0.1	0.025	0.01
	none	0	0	0	0

Figure A. Intermediate data derived in development of non-commercial fisheries catch layers. (1) Map of shoreline accessibility based on the average slope of the shoreline calculated from USGS 10 m DEM and classified into three accessibility categories: high accessibility (e.g., flat, sandy beach environment), low accessibility (e.g., rugged coastal environment with intermediate access), and no accessibility (e.g., high sea cliffs with no access). (2) Map showing presence of roads within 500 m of the shoreline by type of road calculated using US Census Bureau TIGER line data. (3) Map showing the over-water distance to active boat harbors and launch ramps with inset graph of the Gaussian decay function applied in order to approximate boat-based fishing intensity.

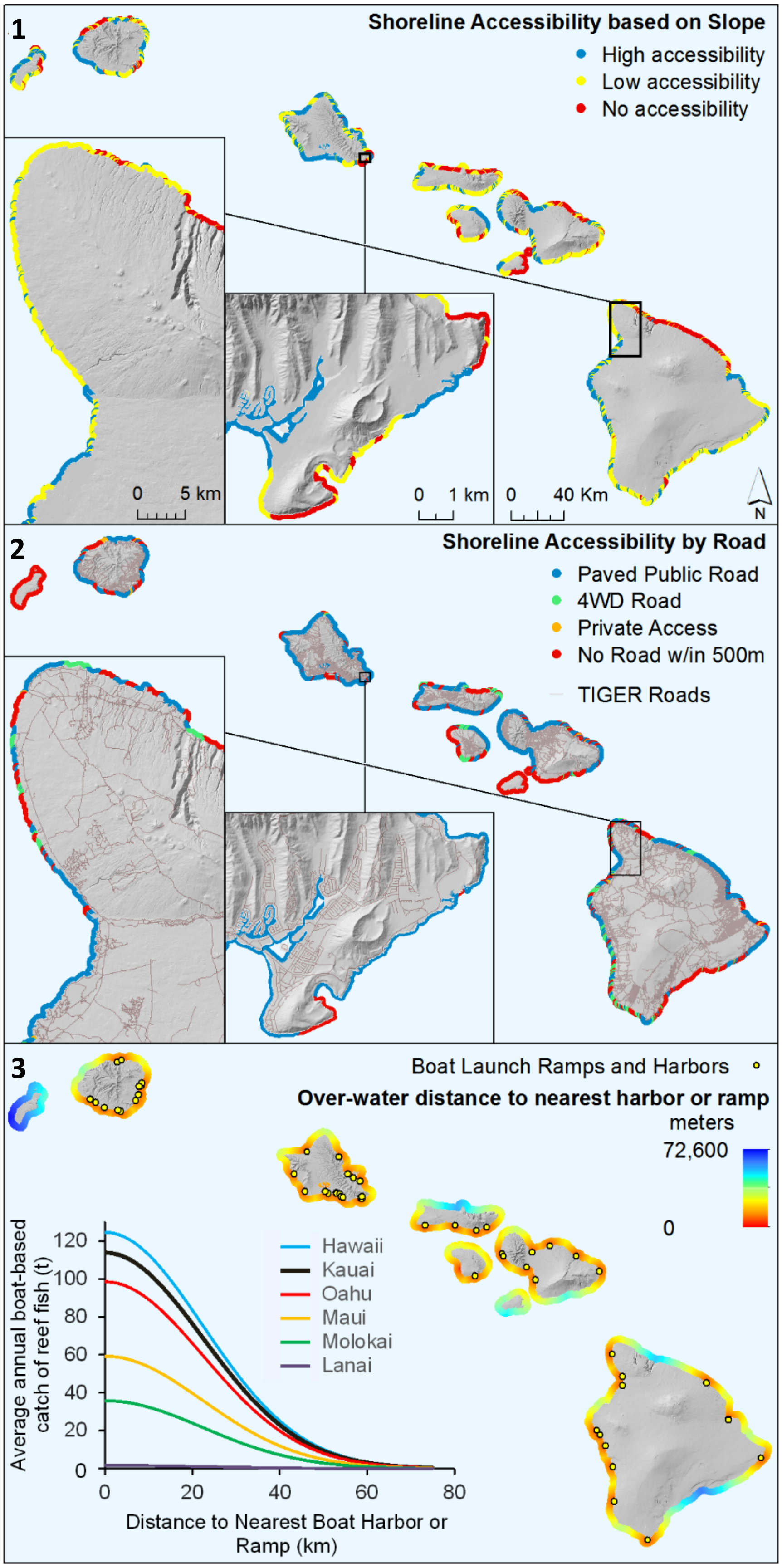

Figure B. Decay function used to decrease spearfishing catch with depth.

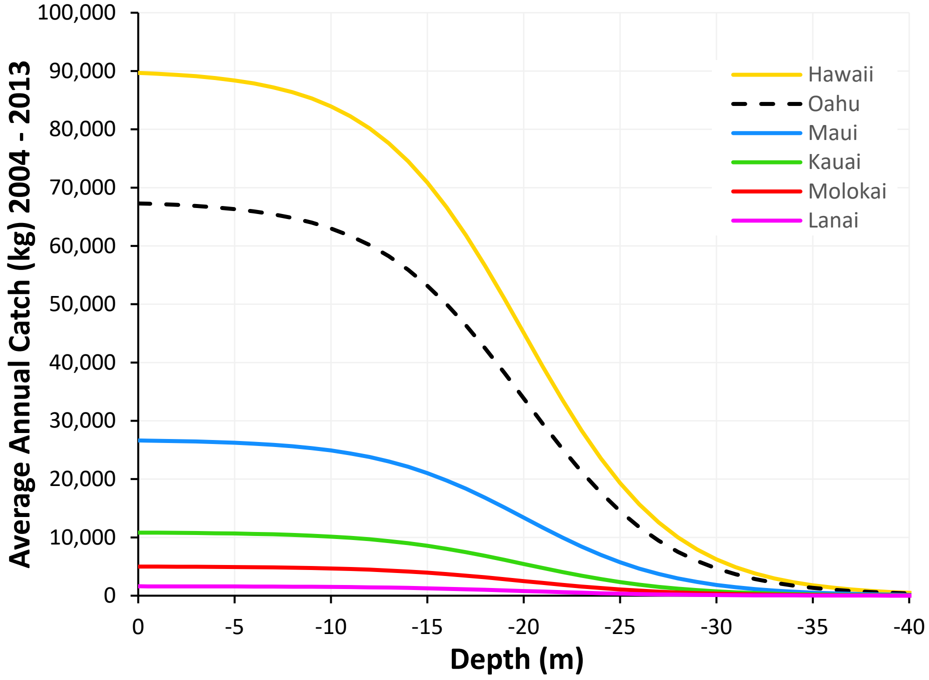

Supplement: S2 Supplement — (PDF) [file pone.0189792.s002.pdf]
